# Refining the Protocol for Equine Umbilical-Cord-Derived Mesenchymal Stem Cell Isolation: A Pilot Study on Resource Efficiency and Cellular Properties

**DOI:** 10.3390/ani16203258

**Published:** 2026-10-16

**Authors:** Annika-Céline Hollender, Hannah J. Stage, Sebastian Rausch, Susanne Trappe, Julius Dahl, Tabea Ockler, Jörg R. Aschenbach, Heidrun Gehlen

**Affiliations:** 1 Equine Clinic, Surgery and Radiology, School of Veterinary Medicine, Freie Universität Berlin, Oertzenweg 19b, 14163 Berlin, Germany; 2 Institute of Veterinary Physiology, School of Veterinary Medicine, Freie Universität Berlin, Königsweg 56, 14163 Berlin, Germany; 3 Institute of Immunology, School of Veterinary Medicine, Freie Universität Berlin, Robert-von-Ostertag-Str. 7, 14163 Berlin, Germany

**Keywords:** equine, mesenchymal stem/stromal cells, Wharton’s jelly, microbial contamination, cost-efficiency, uc-MSCs, non-inferiority, umbilical cord, processing time

## Abstract

Equine mesenchymal stem cells (MSCs) are vital tools for preclinical studies and regenerative medicine, due to their multipotent nature and immunomodulatory potential. Wharton’s jelly provides a non-invasive and abundant source of MSCs. However, the isolation from equine umbilical cord remains challenging, as tissue decontamination is difficult and the enzymatic isolation methods often yield variable amounts of cells. In addition, the established protocols are frequently labour-intensive, time-consuming, and costly. In this pilot study we adapted existing protocols with the aim to improve contamination rates, time commitment and costs. A thorough decontamination method and increased collagenase I digestion time from 3 h in the reference method (RM) to 24 h for the novel method (NM) were employed. Trilineage differentiation, RT-qPCR and flow cytometry were used to characterise both groups. Finally, a non-inferiority analysis was performed to confirm the feasibility of the NM. The changes in protocol led to the decrease in overtime and nightshifts, a faster postisolation expansion by reducing the culture duration by 13.5 ± 11 d, and minimised overall contamination rates (6.3% across 64 samples; RM = 3.1%, NM = 9.4%) compared with most published protocols. Consequently, we were able to reduce the costs per sample by 21 ± 21%.

## Simple Summary

Mesenchymal stem cells (MSCs) are unique cells that can develop into multiple different cell types like cartilage (chondrocytes), bone (osteoblasts), and fat cells (adipocytes). They are able to self-renew and are versatile tools for regenerative medicine, tissue engineering, and preclinical drug studies. The collection of MSCs from the equine umbilical cord is a non-invasive method, as it utilises material normally classified as biological waste tissue. However, working with the tissue surrounding the large blood vessels of the umbilical cord, the so-called Wharton’s jelly, poses several challenges, for instance, the possibility of variable cell yields after isolation, cell culture contamination with bacteria or fungi, night- and overtime labour and high costs. To tackle these obstacles, we adapted existing protocols regarding decontamination measures and the processing time within the specialised enzyme solution “collagenase I”, that breaks down tissue and removes cells from the Wharton’s jelly. In this pilot study, we were able to develop a method that resulted in shorter cell culture duration and lower contamination rates of cultures in comparison to most established protocols. Furthermore, it reduced the costs per sample and number of required nightshifts. As a next step, these findings should be verified utilising a larger sample size.

## 1. Introduction

Mesenchymal stem cells (MSCs) offer versatile applications in vivo and in vitro across regenerative medicine, tissue engineering, and preclinical drug screening [1,2,3,4,5,6,7,8].

Equine MSCs were first isolated from bone marrow in 1998 by Fortier et al. [9]. Since then, a plethora of methods have been developed for obtaining equine MSCs from several types of adult and foetal tissue, including, but not limited to, adipose tissue [10], peripheral blood [11], hair follicles [12], synovial fluid/membrane [13,14], muscle [15], tendon [16,17], endometrium [18], amniotic membranes [19], amniotic fluid [20], umbilical cord blood [21], and umbilical cord matrix [22].

MSCs derived from umbilical cord matrix (uc-MSCs) play an increasing role in equine sports medicine, on the one hand commercial veterinary cell products like HorStem^®^ (EquiCord SL, Madrid, Spain) can be used to treat mild to moderate degenerative joint disease in horses and on the other hand the capability of uc-MSCs in treating skin wounds [23,24] and osteoarthritis [25] is currently being studied.

Contrary to most approaches, uc-MSCs can be obtained non-invasively and in abundance. Multiple protocols with varying success rates from 33% [26] to 100% [27] have been published in the past 20 years for uc-MSCs.

Even after choosing the tissue origin, multiple different paths become available for isolation and culture: explant techniques, enzymatic digestion of tissues or the type of cultivation medium and additives, just to name a few [26,28,29].

However, when looking at all those protocols, some things stand out that are common to all: the isolation of equine uc-MSCs is time-consuming and expensive [30,31,32]. Furthermore, issues like unpredictable cellular yields [33] and a high risk of contamination stand out [20,27,34]. Burk et al. reported a contamination percentage of 16.7% [17]. Denys et al. screened for a range of equine-specific pathogens and found 47.4% of their samples to be contaminated with at least one pathogen [34]. Across multiple studies, uc-MSCs have been found to bear a higher risk of contamination, ranging from 0% [27] to 66.6% affected samples [26].

In order to address these four main issues, we set out to improve existing protocols [17,27]. For the enzymatic isolation of equine uc-MSCs, cells are harvested from the umbilical cord matrix, more specifically perivascular Wharton’s jelly [35,36], directly after parturition. We chose foetal stem cells due to their potentially more multipotent nature [37] and Wharton’s jelly (WJ) because it can easily be obtained in larger quantities; the sampling process after birth is non-invasive, without interrupting the crucial bonding moments between mare and foal [38,39]. Wharton’s jelly has been proven to yield an up to 222-fold higher cell count than umbilical cord blood [17] and especially the perivascular region of WJ contains more than 45% of all cells in human WJ, which makes it a convenient source of MSCs [36,40].

Typical procedures for enzymatic isolation utilise digestion durations from 1 [41] to 20 h [31]. Aside from labour costs, the expenses for materials and reagents add up fast, especially foetal bovine serum (FBS) and collagenase I are costly, as has been discussed in the past by several authors [30,42].

To combat contamination, we chose to build on the protocols established by Passeri et al. [27] and Burk et al. [17]. Passeri et al. showed that a 24 h soak of the tissue in antimicrobial solution decreased the risk of contamination significantly to 0% in five samples tested [27]. However, this resulted in another issue as it added one more day to the protocol instead of saving time. We countered this by adapting the disinfection time to 12 h with previously used antimicrobials [27] but additionally adding gentamycin [17].

The aim of our study was set to compare two isolation methods, the reference method (RM) with 3 h of collagenase I incubation [33] and the novel method (NM) with 24 h of digestion.

With the NM we created a work break and made sure that all lab work can be performed during regular working hours. A similar method was established by Corradetti et al., where umbilical cord tissue was stored in 4 µg/mL amphotericin B, 100 IU/mL penicillin and 100 µg/mL streptomycin in saline solution for a maximum of 12 h; however, neither the exact time of storage nor the percentage of contaminated samples was stated in that publication [43].

Extracting the exact collagenase I activity and concentration from the literature was complicated, as most protocols use % or mg/mL as units; however, U/mL or U/mg would be more appropriate, as the concentration between batches might vary and therefore interfere with reproducibility [44]. Other protocols used between 0.4 mg/mL [22] and 2.4 mg/mL [17,30], however, the mL per gram of tissue was rarely stated [20,28]. We chose 500 U/mL collagenase I (2.0 to 2.5 mg/mL).

In summary, this pilot study was aimed at exploring a modified isolation and cultivation protocol (NM) and measuring its effects on cost of material, time commitment, contamination risk and cellular properties of uc-MSCs compared to the reference method (RM).

We hypothesised that the NM matches the cellular properties and contamination rates of the RM while reducing material costs and time commitment.

## 2. Materials and Methods

A detailed list of all materials used can be found in Supplementary Tables S1–S5.

### 2.1. Collection and Cleaning

Umbilical cords were collected from *N* = 32 warmblood mares and foals in one stud farm. The foals’ sex and whether they were carried by a surrogate mare via embryo transfer or by their mother were considered as best as possible before sampling; however, depending on the timing of foaling, even allocation to the groups was not always possible. Therefore, half of all samples taken were embryo transfer foals from surrogate mares and half were natural pregnancies. Of samples taken, 57% were from colts and 43% from fillies. The mares’ ages averaged at 9.8 ± 5 years. Samples were taken in accordance with the German animal welfare legislation (see section Institutional Review Board Statement; reference number 7221.3-14163_19306_23). Mares and foals were included in the study after a physiological pregnancy and parturition. Samples were collected after complete foetal membrane expulsion in order to not interrupt the mare–foal bonding.

Umbilical cord samples of 20 cm were collected from the allantoic portion near the placenta (as defined previously [45]), avoiding areas damaged during or after birth. The samples were cut with sterile scalpel blades and immediately cleaned under running 4 °C tap water for 5 to 10 min, until macroscopically clean.

Afterwards, the samples were disinfected [17], with some protocol adaptations. First, the samples were submerged in 70% ethanol (Carl Roth GmbH + Co. KG, Karlsruhe, Germany) for 3 min, constantly agitated to ensure even coverage in ethanol of all crevices; next, they were air-dried and then submerged in 1% iodine solution (B. Braun Vet Care GmbH, Tuttlingen, Germany) for 3 min, again constantly kept in motion. Finally, the samples were thoroughly rinsed in sterile phosphate buffered saline without Ca^2+^ and Mg^2+^ (PBS (−/−); Thermo Fisher Scientific Inc., Waltham, MA, USA) until the solution was clear. Each sample was then placed in a sterile bag and submerged in 500 mL of sterile buffered transport solution as developed by Passeri et al. [27] and modified by Burk et al. [17], containing PBS (−/−) with 500 IU/mL penicillin, 0.5 mg/mL streptomycin (Sigma-Aldrich^®^, Merck KGaA, Darmstadt, Germany), 2.5 μg/mL amphotericin B (Corning Inc., Corning, NY, USA) and 0.05 mg/mL gentamycin (Carl Roth GmbH + Co. KG, Karlsruhe, Germany) (5% Pen/Strep, 1% Amp B, and 0.1% Genta). The bag was sealed airtight, double bagged, and then heavily agitated to ensure that all tissues were evenly and completely submerged in antimicrobials. The samples were then stored overnight at 4 °C for 12 h to reduce microbial contamination; overnight storage time was half the duration of the original protocol [27].

### 2.2. Tissue Preparation and Enzyme Digestion

After 12 h, samples were processed further by being thoroughly rinsed in a fresh sterile transport solution, isolating vessels, and preparing the tissue (Figure 1b,c) as previously published [43,46]. The perivascular Wharton’s jelly was cleaned from vessels, intervascular Wharton’s jelly, and endothelium and rinsed again. Tissue samples were finely minced with sterile scissors into 1–3 mm^3^ cubes, then 10.5 ± 1.0 g were transferred into 50 mL centrifuge tubes and submerged in enzyme solution (2 mL/g). The enzyme solution was composed of low-glucose DMEM (Thermo Fisher Scientific Inc., Waltham, MA, USA) and collagenase I (Thermo Fisher Scientific Inc., Waltham, MA, USA) at 500 U/mL (2 to 2.5 mg/mL, depending on the batch of collagenase I) [17,33]. For each pair (RM/NM), samples were digested using the same batch of collagenase I. The tubes were set in a water bath at 37 °C for 3 h (RM) or 24 h (NM) and vortexed regularly.

After the incubation period, all samples were first filtered through 2 mm sterile metal mesh (Fackelmann GmbH Co. KG, Hersbruck, Germany), rinsed with PBS (−/−), centrifuged (600× *g*, room temperature, 10 min), and filtered through a 100 μm cell strainer (Corning Inc., Corning, NY, USA), then rinsed and centrifuged again. After one final round of washing, the cell pellet was resuspended in the basic cell culture medium, containing low-glucose DMEM, 20% foetal bovine serum (FBS) (Sigma-Aldrich^®^, Merck KGaA, Darmstadt, Germany), 1% Pen/Strep, 1% Amp B and 0.1% Genta [17]. However, we increased the Amp B concentration to 1% instead of 0.2% to account for the reduced antifungal activities in the presence of larger serum quantities [47] and seeded cells into one T75 cell culture flask (TPP Techno Plastic Products AG, Trasadingen, Switzerland).

A total of 64 samples of cells were collected from the same segment of perivascular Wharton’s jelly tissue to compare both digestion methods. To minimise individual-dependant bias, 3 h (RM) and 24 h (NM) samples were always taken from the same foals for paired comparison.

### 2.3. Experimental Design

The RM and NM for equine uc-MSC isolation were compared using a paired, non-inferiority framework for this pilot study. All samples were decontaminated and disinfected in one piece and tissue was allocated to each method after mincing to ensure baseline comparability (Figure 2).

### 2.4. Cell Culture

The cells were cultured at 37 °C with 5% CO_2_ in a vapour-saturated incubator. Twenty-four hours after seeding, cells were washed three times with PBS with Ca^2+^ and Mg^2+^ (PBS (+/+); Carl Roth GmbH + Co. KG, Karlsruhe, Germany) and then further cultured with medium replacements and microscopic checks twice per week until 80–90% confluence. For passaging, cells were detached with 0.25%/0.02% trypsin/EDTA (Thermo Fisher Scientific Inc., Waltham, MA, USA) for 10 to 15 min and split 1:3; they were then frozen after passage 2 (P2) in cryopreservation medium containing low-glucose DMEM, 40% FBS, 10% dimethyl sulfoxide (DMSO) (AppliChem GmbH, Darmstadt, Germany), 1% Pen/Strep, 1% Amp B, and 0.1% Genta, modified as published before [29,47,48] at 2 million cells per mL in Mr. Frosty™ (Thermo Fisher Scientific Inc., Waltham, MA, USA) at −80 °C for at least one day and then in liquid nitrogen for permanent storage.

The isolation success was scored by microscopy (MOTICEUROPE, S.L.U., Barcelona, Spain) [48], with either 0 points for no cell attachment and 1 point for cell attachment before passage 1 (P1). In addition, within the first microscopic examination after isolation the number of cells attached was scored; 0 points for no cells, 1 point for single cells within the average field of view (FOV), 2 points for small island formations with up to 10 cells, and 3 points for larger islands with more than 10 cells per island. Contamination was scored separately; if the cell culture was clean until the first passaging after seeding, it was considered “not contaminated” with a score of 0; if a fungal or bacterial contamination appeared within passage 0 (P0) after seeding, it was counted as contaminated with a score of 1, due to the initial tissue being contaminated. Contamination detected after the first passaging was not taken into consideration. As the RM and NM protocols were compared in a paired study design, both samples from that donor were excluded from subsequent experiments whenever one of them was contaminated and therefore discarded.

### 2.5. Characterisation

All samples were characterised as first suggested by the International Society for Cellular Therapy (ISCT) [49] and then further specified for equine stem cells by De Schauwer et al. [50] and Guest et al. [51] with a modified flow cytometry panel. Out of the remaining 28 paired samples, 8 were lost due to contamination in later passages after an autoclave failure and 5 more after technical cryostorage malfunctions, leading to only 15 of 32 paired samples usable for flow cytometry and RT-qPCR (*N* = 15). All further characterisation experiments, including trilineage differentiation and cell proliferation assays, were conducted using paired samples of three foals (*N* = 3) in P4 after cryopreservation.

The cells were examined for fibroblast-like growth patterns and spindle-like shape as well as their ability to adhere to the plastic culture vessel.

### 2.6. Inter-Passage Time and Population-Doubling Time

To compare both methods, the culture duration between passages in days (inter-passage time (IPT) in d) for *N* = 20 paired samples was calculated from isolation until cryopreservation, including the mean and standard deviation per method and passage (P0 to P1; P1 to P2; P2 to cryopreservation). For three paired (*N* = 3) samples in P4, a population-doubling assay was performed [52,53]. In short, 30,000 cells/well (3.5 cm^2^) were sown into 12-well plates on day zero (T_0_), and each day three wells per sample were harvested and counted in duplicate with the Automated Cell Counter (Bio-Rad Laboratories GmbH, Feldkirchen, Germany). The settings were previously established by Stage et al. [53] and confirmed for this type of primary cells via manual counting in the Neubauer chamber. The percentage of dead cells was also counted, and the means were calculated for both values. This was carried out until day 8 (T_8_).

The calculations were carried out utilising Equation (1) [52], where T is the harvest time of culture, T_0_ the starting time of culture, and N_T_ and N_0_ are the cell count of harvest and cell count of seeding, respectively.

(1) $$\mathrm{PDT}=\frac{\left(T-T_{0}\right)*\mathrm{lg}2}{{\mathrm{lgN}}_{T}-{\mathrm{lgN}}_{0}}$$

**Equation (1).** Population-doubling time (PDT) in hours [52].

### 2.7. Trilineage Differentiation

All differentiation trials were executed in duplicates (chondrogenic induced samples) or triplets (all other samples), using the same three paired samples (*N* = 3) as for the PDT. All culture vessels for the adipogenic and osteogenic differentiations were coated with 2 µg/cm^2^ of laminin (Corning Inc., Corning, NY, USA) to reduce cell detachment, as previously established [53]. Negative controls (NCs) were included as triplets at 20,000 cells/cm^2^ density in laminin-coated 24-well plates (Starlab GmbH, Hamburg, Germany).

#### 2.7.1. Chondrogenic Differentiation

Chondrogenic differentiation was induced in 3D pellet culture as published before [53,54]. In brief, cells were thawed, expanded, and passaged and 5 × 10^5^ cells were centrifuged into a pellet. The cells were then seeded into uncoated 15 mL centrifuge vials (TPP Techno Plastic Products AG, Trasadingen, Switzerland), covered with 2 mL of chondrogenic differentiation medium (CD medium) [53], and vortexed for 1 min every day. The CD medium was replaced three times per week; the caps were not screwed on tight to ensure gas exchange in the incubator. The culture was continued until day 21 (T_21_), when the pellets and the NCs were fixed in 4% formalin (Carl Roth GmbH + Co. KG, Karlsruhe, Germany), the pellets were then embedded in paraffin (A. Menarini Diagnostics, Berlin, Germany) and cut for histological examination (Thermo Fisher Scientific Inc., Waltham, MA, USA).

Alcian blue (Morphisto GmbH, Offenbach am Main, Germany) and haematoxylin–eosin stains (Waldeck GmbH & Co. KG, Münster, Germany) were chosen as established in-house [53]. Stained slides and NCs were scored blinded with the Bern score by Grogan et al. [55], modified for Alcian-blue-stained samples [30,53]. Samples scored 0 to 3 points for uniformity as well as matrix and cell morphology, for a maximum of 9 points in total.

#### 2.7.2. Osteogenic Differentiation

Osteogenic differentiation was induced as established in-house [53]. In short, cells were thawed, expanded, passaged and 20,000 cells/cm^2^ were seeded in triplets into laminin-coated 24-well plates. The cells were expanded until confluence, covered with 1 mL of osteogenic differentiation medium (OD medium); the medium was changed three times per week. The culture was continued until T_21_, when cells were fixed in 4% formalin.

Von Kossa staining (Morphisto GmbH, Offenbach am Main, Germany) and nuclear fast red stains were performed for extracellular calcium deposits and nuclei [53]. The samples were evaluated via microscope and photographs were examined in a blinded trial.

A semi-quantitative scoring system (Table 1) for the evaluation of osteogenic differentiation success and von Kossa staining was developed based on other scoring systems [30,55]. Samples scored 0 to 3 points for coverage of field of view (FOV) by mineral deposits in %, 0 to 3 points for the size of the predominant variety of deposits in relation to cells, since FOVs usually contain a mix of granule sizes, and 0 to 3 points for the arrangement and clustering of mineral deposits, all measured with 10× magnification. In total, a maximum of 9 points can be achieved. The implementation of this system was necessary to keep osteogenic differentiation assessable even if plate reader scoring [56] or ImageJ scoring [57] could not be completed due to technical restrictions or overgrowth of the negative controls.

#### 2.7.3. Adipogenic Differentiation

As specific media components for the established in-house protocol were not available at the time of the experiment, the established protocol was replaced with the Stem Cell Kit for adipogenic differentiation with some added modifications [54]. In short, cells were thawed, expanded, and passaged and 20,000 cells/cm^2^ were seeded in triplets into laminin-coated 24-well plates. Cells were expanded until confluence, covered with 1 mL of adipogenic differentiation medium (AD medium), consisting of a StemPro™ Adipogenesis Kit (Thermo Fisher Scientific Inc., Waltham, MA, USA) supplemented with 5% rabbit serum (Thermo Fisher Scientific Inc., Waltham, MA, USA), 1% Pen/Strep, 1% Amp B and 0.1% Genta. The medium was changed every other day; the culture was continued until day 7 (T_7_), when cells were fixed in 4% formalin.

Nile red and DAPI staining was executed as established in-house [53]. Blinded scoring was then performed as described [30,54], scoring 0 to 3 points for the percentage of differentiated cells and 0 to 3 points for the size and arrangement of lipid droplets.

### 2.8. Reverse-Transcription Quantitative PCR

The cells for reverse-transcription quantitative PCR (RT-qPCR) were detached using Accutase^®^ (Carl Roth GmbH + Co. KG, Karlsruhe, Germany) to keep them suitable for flow cytometry as well as RNA extraction. One T75 flask of cells in P3 was rinsed with PBS (−/−) and then incubated with 10 mL of Accutase^®^ according to the manufacturer’s instructions. RNA extraction (Macherey Nagel GmbH & Co. KG, Düren, Germany), cDNA synthesis (Bio-Rad Laboratories GmbH, Feldkirchen, Germany), and SYBR Green (Bio-Rad Laboratories GmbH, Feldkirchen, Germany) RT-qPCR were executed following manufacturers’ instructions, using the ViiA7 Thermocycler (Applied Biosystems/Life Technologies; Foster City, CA, USA) and identical primers plus an additional primer (Table 2) for the cell surface antigen CD90 (*THY-1*). *THY-1* was established using the protocol established in-house [48]. Glyceraldehyde-3-phosphate dehydrogenase (*GAPDH*), β-actin (*ACTB*), and 18S ribosomal RNA (*RN18S*) were employed as housekeepers and POU class 5 homeobox 1 or octamer-binding transcription factor 4 (*POU5F1/OCT4*), DNA methyltransferase 3 beta (*DNMT3B*), DENN domain containing 4A (*DENND4A*), MYC proto-oncogene, BHLH transcription factor (*MYC*), and the Thy-1 cell surface antigen CD90 (*THY-1*) were analysed as stemness-associated transcriptional markers. The data were normalised to the three housekeeping genes (ΔCt). All statistical analyses were performed using ΔCt values. Statistical testing of ΔCt values was only performed if the Ct values of the respective samples were more than four cycles apart from the average Ct values of H_2_O and no-reverse-transcriptase controls (–RT controls).

### 2.9. Flow Cytometry

All of the samples were examined for the entire surface marker panel as previously established [48]. In short, cells were detached using Accutase^®^, washed, and counted. Then, 1 × 10^5^ cells were stained in V-shape 96-well plates (Greiner Bio-One, Kremsmünster, Austria). Primary antibody staining was performed for CD45, CD34, CD29, CD44 and CD105 (Table 3). Secondary antibody for CD45 was Streptavidin-Alexa Fluor 488 and cell viability was monitored using viability dye in eFluor 506 (eF506). Cells were acquired using the BD FACSCanto II (BD Biosciences, Milpitas, CA, USA) and analysed using the FlowJo Software (Version 8; BD Biosciences, Milpitas, CA, USA). The antibody for CD90.1, Clone OX-7 in Alexa Fluor 450 utilised by Trachsel and Stage et al. [48] was tested as well as two other CD90.1 antibodies and one CD90.2 clone. The antibody panel was adapted with a matching viability dye in eF506. New antibodies were assessed using leftover cell suspensions from bronchioalveolar lavages of equines and leftover EDTA blood samples collected during routine diagnostics in the Equine Clinic of Freie Universität Berlin. Gating was performed to exclude subcellular debris with low forward light scatter (FSC) and low sideward light scatter (SSC). After removal of potential doublets based on FSC properties, dead cells were excluded using the viability dye (Supplementary Figure S1).

### 2.10. Cost-Efficiency Analysis

The catalogue prices in euros for all media components necessary for the isolation and cultivation of uc-MSCs were acquired through the manufacturers’ retail websites [58,59,60,61,62,63,64,65,66,67], with settings and taxes for Germany and no discounts applied (see Table A1). All further calculations were conducted using the RM as the baseline, with the NM as the relative difference. Results are therefore reported as percentages (mean ± standard deviation); whereas the prices, currencies, and taxes may vary depending on the economic region as well as supply and demand, the difference between the two methods does not.

Defining the price of labour is difficult, since hourly wages differ vastly depending on country, employee, and employer. As a reliable and transparent data baseline for this publication we therefore chose the gross wages in euros defined for personnel employed by the German government. The *Tarifvertrag für den öffentlichen Dienst* (TVöD) [68] and the *Tarifvertrag für den öffentlichen Dienst der Länder* (TV-L) [69] include clear regulations regarding over- and nighttime pay and a salary scale for employees of state institutions. Specifically, the pay grades (*Entgeltgruppen*) E5 to E14 of laboratory personnel at the basic experience level 1 were used (see Table A2) and the median was calculated. As discussed above, all further results are presented as the relative difference between the RM (baseline) and the NM.

Critical time points during the protocol process were recorded, times were linearised and centred around midnight to account for the circular nature of time-of-day data and prevent distortion of results. The median (5th percentile; 95th percentile) was then calculated, using midnight as the central reference as equine births usually occur around midnight [70].

### 2.11. Statistical Analysis

All values given are means ± standard deviations unless stated differently. All flow cytometry data are presented as medians (IQR; 25th–75th percentile).

Normality of the data was visually assessed prior to analysis, using a Q-Q plot. To compare the 3 h method (RM) with our extended 24 h incubation (NM), a paired *t*-test for non-inferiority analysis was performed with *α* = 0.05, using NCSS 2026 statistical software (NCSS, LLC. Kaysville, UT, USA) [71,72,73,74]. Technical replicates were averaged to a single value per donor before analysis, each donor was handled as the experimental unit (*N* = donors). All further paired tests were performed with these averaged values. The aim of the present study was to develop a method that is not inferior in the categories inter-passage time (IPT), population-doubling time (PDT), trilineage potential, and gene expression. Since the immunophenotype data after flow cytometry were skewed either to the right or left, we performed the non-inferiority analysis using the non-parametric Wilcoxon signed-rank test with continuity correction and *α* = 0.05. For all ordinal data from the trilineage assays, differentiation scores were likewise assessed using the one-sided Wilcoxon signed-rank test on donor-level paired differences; exact *p* values are reported where computable and the normal approximation with continuity correction was used in all other cases (i.e., in the presence of ties). Paired effect estimates and corresponding confidence intervals for non-parametric data were calculated as the Hodges–Lehmann estimate, with 95% confidence intervals (wilcox.test, conf.int = TRUE, R, Supplementary Listing S1).

The *p* values from the non-inferiority tests for IPT, PDT, and trilineage potential were corrected for multiple testing using the Holm method (p.adjust, R, Supplementary Listing S2) at a familywise α of 0.05. Calculated *p* values for the RT-qPCR and flow cytometry results (five markers each) were also corrected using the Holm method at a familywise α of 0.05.

In summary, the null hypothesis for each category was that the NM is not non-inferior to the RM. The alternative hypothesis was that the NM is non-inferior to the RM (Table 4). Results were considered statistically significant at *p* < 0.05.

Analyses performed on *N* = 3 donors are considered exploratory, whereas the assays with larger sample sizes provide more robust evidence; nevertheless, all findings require further validation.

Generally, the margins of non-inferiority for each test were set pragmatically in such a way that the performance of the NM uc-MSCs could still be tolerated, ensuring study-specific fitness-for-purpose rather than reflecting biologically or clinically validated equivalence limits. Different aspects were taken into account to ensure the NM uc-MSCs could still fulfil their purpose in this in vitro pilot study, including tolerable limits of heterogeneity of the cultured cell population, reduction in differentiation potential, and stemness-associated transcriptional markers and antigen effects (Table 4). More specifically, for the downstream in vitro studies planned, a population of cells containing 15% more non-target cells was prespecified as acceptable for the gene expression and immunophenotype, as this variation remains within passage- and donor-dependent variation normally present in primary cell cultures (Equation (2)).

ΔCtmax = −log2(0.85) ≈ 0.2345 (2)

**Equation (2).** Prespecified non-inferiority margin for RT-qPCR data analysis.

Similarly, a slower PDT (up to 12 h) and a longer IPT (up to 7 d) were prespecified as acceptable, as differences of this magnitude lie within donor-, passage-, and seeding-density-dependent variation in expansion kinetics. For this pilot study, it was the operative requirement that the cells reach a sufficient quantity within one extra week to ensure that applications within the same differentiation assays remained comparable. Finally, a reduction of 0.5 points per subcategory while scoring differentiation success, resulting in a 1-point (adipogenic) or 1.5-point (chondrogenic, osteogenic) decrease, was prespecified as acceptable, as a reduction of this magnitude corresponds at most with a modest reduction in proportion of differentiated cells.

## 3. Results

The data presented followed a normal distribution, which is consistent with the nature of the variables measured; except the flow cytometry results that were left-skewed for the positive markers and right-skewed for the negative markers due to strong ceiling or floor effects, respectively. Additionally, the labour cost data did not follow a normal distribution pattern.

### 3.1. Isolation

The cell suspension after the rinsing cycles of the isolation protocol remained heavily interspersed with erythrocytes; in both methods, but especially the NM, cells showed as larger aggregates under the microscope, although cell strainers were utilised.

The total isolation success rate for all samples was 100% for RM (3 h) and 100% for NM (24 h). Taking the microbial contamination into account, the total rate of successful cultures was 93.8% for all 64 samples. The 3 h RM had a success rate of 96.9% and the 24 h NM had a success rate of 90.6%. Microbial contamination within P0 was observed in 3.1% of the RM and 9.4% of the NM samples, and therefore in a total of 6.3% across all 64 samples. In all cases of contamination, only one of the paired samples was affected, either in the NM or RM group. Results were concordant in 28 of 32 paired samples. Due to the low number of discordant pairs (*N* = 4), a paired statistical analysis (McNemar test) was not performed. As this test depends solely on discordant pairs, it would have been underpowered, with a minimum attainable two-sided p value of 0.125. The results of the contamination analysis are therefore presented and compared descriptively.

Samples classified as contaminated showed a sudden culture medium colour change indicating a pH shift and macroscopically cloudy medium together with microscopic detection of coccoid organisms on inverted light microscopy. Neither macroscopically visible fungal growths nor microscopic hyphae were observed.

### 3.2. Cultivation

#### 3.2.1. Inter-Passage Time

In both approaches, first cells attached within 24 h; however, the NM scored 2.69 ± 0.54 points compared to 1.47 ± 0.57 points in the RM. Other studies showed first attachment after 14 d [26]. On average, our RM cells were first passaged after 13.8 ± 2.4 d with an inter-passage time between P1 and P2 of 14.7 ± 4.3 d and P2 and P3 of 12.6 ± 4.7 d. The NM cells showed shorter IPTs on average for every passage: P0–P1 11.5 ± 2.5 d, P1–P2 7.55 ± 3.6 d and P2–P3 8.6 ± 3.3 d (Figure 3). Therefore, the NM IPT was on average 2.35 d shorter during P0, 7.1 d in P1 and 4.0 d in P2. In total, the RM took 41.1 ± 9.2 d from isolation to the end of P2, whereas the NM took 27.6 ± 6.6 d, thus showing a difference in culture duration of 13.5 ± 11 d. The NM met the prespecified criterion for non-inferiority within the predefined margin for all three passages (+7 d), resulting in *p* < 0.001. Further statistical data, including the paired effect estimates, are provided in Supplementary Table S6.

#### 3.2.2. Population-Doubling Time

The population-doubling assay showed the cells of both the RM and the NM group entering the log phase of growth after a brief one- to two-day lag period, no plateau phase was reached within the eight days of culture, but the growth was limited due to confluency being reached (Figure 4). The samples of the NM showed a quicker adaptation to growing conditions in the form of exponential growth as they doubled in amount from day 1 to 2 (5.4 × 10^3^ ± 603 cells to 12.0 × 10^3^ ± 2309 cells), whereas the RM cells needed another day to reach exponential growth (6.9 × 10^3^ ± 1893 cells to 8.3 × 10^3^ ± 3899 cells).

The PDT (calculated during exponential growth from T_1_ to T_6_) was 23.2 ± 2.7 h for the RM and 20.1 ± 2.2 h for the NM. The NM showed a faster growth on average by 2.7 h per doubling. The NM met the prespecified criterion for non-inferiority within the predefined margin (+12 h), resulting in *p* = 0.045. Further statistical data, including the paired effect estimates, are provided in Supplementary Table S7.

### 3.3. Characterisation

Cells were spindle-shaped with fibroblast-like growth patterns while grown in a monolayer adhered to plastic. Once confluent, 3D clusters of spheroid shape formed, and after trypsinisation and reseeding the cells were spindle shaped again. Initially the cells resembled the rapid self-renewing, spindle-shaped, and flattened cell groups (RS, SS, and FC groups) developed by Haasters et al. [75] but shifted towards more uniform spindle-shaped cells with increasing confluence and completely after the first passage at the latest; no differences could be observed between the RM and NM (Figure 5).

#### Trilineage Differentiation

Chondrogenic differentiation was performed successfully in all tested samples, evident by the positive Alcian blue staining indicating glycosaminoglycan presence within the extracellular matrix (Figure 6). The RM scored 7.97 ± 1.00 points and the NM scored 7.78 ± 0.97 points in the modified Bern score [55]. The NM did not meet the prespecified criterion for non-inferiority within the predefined margin (−1.5 points), resulting in *p* = 0.375 when compared to the RM.

Osteogenic differentiation was observed in all samples regardless of the isolation method, demonstrated by strongly positive von Kossa staining with the formation of dense, dark brown to black silver phosphate precipitates (Figure 7). These deposits were distributed throughout the culture vessels but accumulated towards the well walls. Extensive overgrowth in the form of 3D cell formations in the negative controls caused non-specific staining artifacts and mechanical dye retention. This and the added density of multiple cell layers caused artificially elevated values in the controls of all samples, rendering accurate photometric readings of the controls impossible. An alternative scoring system for semi-quantitative microscopy was developed and employed. The RM scored 5.35 ± 2.02 points and the NM scored 6.01 ± 1.93 points in the osteogenesis score. The paired non-inferiority could not establish non-inferiority by meeting the prespecified margin (−1.5 points, *p* = 0.375).

Adipogenic differentiation was performed successfully in all tested samples, evident by the positive Nile red staining indicating the accumulation of numerous small to large lipid droplets adjacent to the cell nucleus (Figure 8). The RM scored 5.1 ± 0.54 points and the NM scored 5.0 ± 0.63 points in the modified score by Gittel et al. [17,30,54]. The paired non-inferiority could not establish non-inferiority by meeting the prespecified margin (−1 point, *p* = 0.375).

When examining donor-level paired data it becomes evident that 2/3 of the samples (donors A and C) yielded higher differentiation scores in the NM; thus yielding the maximum possible signed-rank statistic (Wilcoxon signed-rank test, exact one-sided *p* = 0.125 before multiple testing correction, the minimum attainable at *N* = 3); given this sample-size-imposed floor, the comparison should be interpreted with caution and is therefore presented alongside the donor-level data below (Figure 9).

### 3.4. RT-qPCR

The spectral photometric measurements performed with the Nanophotometer™ and the Bioanalyzer-Nano™ confirmed the high quality and quantity required for every RNA sample (concentration ratio close to 2 and RIN value close to 10) [48]. All percentage values given are the mean difference of the NM to the RM group; mean ± standard deviation of ΔCt values are displayed in Table 5.

The genes *DNMT3B*, *DENND4A* and *MYC* met the prespecified criteria for non-inferiority, yielding *p* values of 0.034, 0.028 and 0.005 respectively. *DNMT3B* expression was increased by 6.9% on average in the NM cells compared to the RM cells. Furthermore, the uc-MSCs showed a 23.3% higher expression of *DENND4A* and an upregulation of *MYC* by 36.3% in the NM compared to the RM group.

*THY1* and *POU5F1/OCT4* were both downregulated by an average of 55.5% and 16.6% respectively and, therefore, the NM did not meet the prespecified margin for non-inferiority (+0.2345 ΔCt, *p* = 1 for *THY1* and *p* = 1 for *POU5F1/OCT4*).

### 3.5. Flow Cytometry

The uc-MSCs expressed markers from the surface marker panel as seen in Table 3. CD90 could not successfully be assessed as a surface antigen, neither clone OX-7 or HIS51 (CD90.1) nor 30-H12 (CD90.2) bound reliably; instead, evaluation at the transcript level via RT-qPCR analysis of *THY-1* was performed (Table 5 and Table 6).

Non-inferiority of the NM uc-MSCs relative to the RM uc-MSCs was not demonstrated in terms of CD29 (*p* = 0.466) and CD44 (*p* = 0.147) expression. However, the NM samples met the prespecified margin for non-inferiority (+15% for negative markers and −15% for positive markers) when it came to the analysis of the negative marker expression of CD34 (*p* = 0.001) and CD45 (*p* < 0.001) and the positive marker expression of CD105 (*p* = 0.015). Overall, the RM uc-MSCs expressed higher percentages of the negative markers CD34 (1.0%, IQR 2.8%) and CD45 (2.5%, IQR 10.4%) compared to CD34 (0.1%; IQR 1.0%) and CD45 (1.5%; IQR 5.4%) expression in the NM uc-MSCs. The positive markers CD29 and CD44 appeared similar when inspecting the median, but when taking the IQR into account, the NM uc-MSCs displayed a greater heterogeneity within their population and did not meet the prespecified margin for non-inferiority (Table 6).

Tested cultures were classified as pure and directly usable for further experiments if they contained >80% CD29- and CD44-positive cells and <5% CD34- and CD45-positive cells. Of all RM samples, 67% passed this threshold and 40% of all NM samples.

### 3.6. Cost-Efficiency Analysis

All displayed costs were calculated based on regional pricing; they may vary depending on the economic and geographic region.

The cost of materials for isolating uc-MSCs from one sample amounts to 60.10 €. The cost per medium replacement is 2.88 € and therefore the cost of cell culture media for the RM (from isolation to the end of P2) is 201.24 ± 43.21 €. The NM produces total media costs of 152.27 ± 25.92 €, therefore reducing the costs by 34.7 ± 27.2%. Taking the contamination rate per group into account, the NM remains economically favourable with 29.8 ± 27.7% reduced costs per sample. The total material costs for samples isolated with the NM amounts to 21 ± 21% less.

The median (min; max) gross wage per hour for state laboratory personnel in Germany is 22.29 € (18.04 €; 31.24 €). Median overtime pay per hour is +30% (+15%; +30%) while nighttime pay (21:00 to 6:00) is fixed at +20%. Overtime at night is compensated with +50% (+35%; +50%) of the regular gross wage per hour.

The foals sampled for this study were born at 23:50 (20:23; 02:36) on day −1 (T_−1_) and usually five to six foals were sampled in one night. Sampling started after foetal membrane expulsion at 0:37 (21:41; 3:11). As shown in Table 7, utilising the NM protocol allowed for the tasks of enzymatic digestion and seeding of cells to be spread across T_0_ and T_1_, instead of the single, long day necessary for the RM. Therefore, the RM protocol takes three days (RM-T_−1_ to RM-T_1_) to complete, however, the shorter total timeframe requires nightshifts and overtime during all three days. In comparison, the NM protocol takes four days (NM-T_−1_ to NM-T_2_), reducing the night shifts and overtime to one unavoidable shift due to the nocturnal time of foaling [70]. In the RM, this leads to additional labour costs of about 11.6% (11.4%; 9.9%) combined compensation for RM-T_0_ and RM-T_1_ per foal. In addition, the employee needs to be compensated with lieu time for the overtime built up and this leads to an additional 13.1% cost (10.7%; 12.6%). In total, the labour costs for the RM are increased per foal by 21.4% (20.9%; 20%) in comparison to the NM.

## 4. Discussion

The primary objective of this study was to achieve a multiparametric optimisation of an existing enzymatic isolation protocol for equine uc-MSCs derived from Wharton’s jelly. We sought to reduce labour and material costs, create a protocol with appropriate break points while reducing nightshifts and finally target contamination issues and variable cellular yields as discussed in the past [20,26,27,30,31,32,33,34].

The adapted protocol for decontamination and disinfection was thoroughly tested across a total of 64 isolations (*N* = 32) from perivascular Wharton’s jelly, using both the RM and the NM. Subsequently, the proliferation potential, differentiation behaviour, immunophenotype, and gene expression profiles were evaluated for further comparison of both uc-MSC method groups. Non-inferiority analyses were performed to establish whether the optimised protocol preserved critical stem cell properties [49,50,51,76] within an acceptable margin of inferiority.

Non-inferiority of cellular properties of the NM uc-MSCs was observed in the present study, apart from *THY-1* (−55.5%) and *POU5F1/OCT4* (−16.0%), as well as CD29 (−3.1%) and CD44 (−0.4%), for which non-inferiority could not be established. The reduced expression of both stem cell genes and surface markers might lead to the assumption that the NM cells were less potent when it comes to multipotency, stemness, and self-renewal [77,78,79,80,81,82]. However, as far as the present study results show, this does not seem to be the case. The prespecified margins for non-inferiority were met by the other stemness-associated transcriptional markers, population-doubling time (PDT), and inter-passage time (IPT). Although the prespecified non-inferiority margins were not met for all three differentiation lineages (*p* = 0.375), examination of the donor-level paired data shows that 2/3 of the samples (donors A and C) yielded higher differentiation scores in the NM. Since the test yielded the minimal *p* value possible for the signed-rank statistic given the sample-size-imposed floor, the comparison should be interpreted with caution and is therefore presented alongside the donor-level data (Figure 9). Since the sample sizes tested were as small as pairs of *N* = 3 for the trilineage differentiation and PDT assays and because both groups—but especially NM uc-MSCs—displayed heterogeneity within the flow cytometry and RT-qPCR results, future testing should include larger groups to further validate the cells stemness. A possible explanation for the larger portion of non-target cells could be that the substantially longer enzymatic digestion dislodges a larger proportion of those unwanted cells; however, this remains to be confirmed. Utilising fluorescence-activated cell sorting (FACS) to account for this type of contamination could address this limitation (see below).

For NM uc-MSCs, a shorter IPT for every passage was measured, with the first samples only starting to converge with the RM group during P2. A true convergence could not be witnessed; several more passages would have been necessary. P0 was 17% faster on average, P1 48.5%, and P2 31.8%, reducing the total culture time from isolation to freezing after P2 by 32.8% (13.45 d) for the NM. Other sources showed inter-passage times between P0 and P1 of 10 ± 3.75 d to 21.5 d [17,26,41].

The population-doubling assay confirmed this observation with a PDT of 23.2 ± 2.7 h for the RM and 20.1 ± 2.2 h for the NM. In addition, the NM cells adapted to culture conditions one day faster, allowing further experiments to begin sooner. In summary, this led to a reduction of 33.4 ± 25.2% in material costs for media replacements per sample alone. However, due to the heterogeneity of the cells cultured, a future investigation using a standardised initial seeding number and larger sample sizes should be employed for verification of these preliminary findings.

Significant cell populations positive for CD105 in either the RM or NM group could not be detected. Although it is commonly recognised as a marker for MSCs [49,50,51,76], several studies have reported its absence or variable expression in equine MSCs [41,50,53,83,84,85,86]. CD90 could likewise not reliably be documented as a surface antigen; only *THY-1* transcript abundance could be assessed.

We were nonetheless able to fulfil the minimal requirements for in vitro studies reported by Guest et al. [51] for both NM and RM cells, as both groups expressed CD29 and CD44 as positive markers for mesenchymal stem cells alongside the other characterisations reported.

In a recent study, alternative antibodies were successfully applied for equine uc-MSCs [32]; testing these antibodies for CD90 and CD105 would be a viable option for protocol improvement. Furthermore, the major histocompatibility complex class II (MHC Class II) was not included in the present immunophenotypic characterisation, as this study was limited to the optimisation of isolation techniques and in vitro characterisation of the resulting uc-MSCs, thus limiting the insight into the potential effect of the NM on immunogenicity of NM uc-MSCs. In in vivo studies, particularly for allogeneic administration, MHC Class II should be added to the negative marker panel [50,51]. MHC Class II can be expressed heterogeneously in equine MSCs, varying between donors and passages [87]. MSCs with low MHC Class II expression are more appropriate for allogeneic in vivo applications [88]. As introduced above, utilising FACS instead of regular flow cytometry should allow cultures to be cleaned up and to become more homogenous and predictable [89,90,91,92,93]. Furthermore, the investigation of the three cell types RS, SS, and FC as proposed by Haasters et al. [75] could be included, since all three types were observed in our samples, regardless of method. By using FACS, a more detailed characterisation and tracking of their respective development and potential would be possible, which was beyond the scope of the present study. When comparing the contamination rates of both groups, 1 out of 32 samples (3.1%) of the RM and 3 out of 32 (9.4%) of the NM samples were affected by microbial contamination; the type of contaminating agent was not analysed further. Other studies described contamination rates of 16.7% to 66.6% [17,20,26,30,32,34] with only one study with a sample size of five reporting 0% contamination [27]. So even though we did record a contamination rate triple the RM rate with the NM protocol, we still improved upon the rates generally obtained with umbilical cord material, achieving a low contamination rate of 6.3% over a total of 64 isolated samples. The adapted decontamination and disinfection protocol [17,27] shows promising potential, since the material itself is contaminated during natural birth, as the vaginal canal of the mare and the stable or field are never sterile [94,95,96,97]. In addition, the foetal membranes are usually spread out on some kind of surface or the floor to check for complete expulsion [98,99,100]. This problem has been discussed by several other authors and many decontamination protocols have been developed [17,20,27,31,43].

A possible explanation for the higher contamination rate of the NM, while none of the paired RM samples showed signs of contamination, may be attributed to the fact that the enzyme solutions lack antimicrobial substances. In comparison to the RM, the tissue spends an extra 21 h at 37 °C, allowing the remaining bacteria to continue multiplying with an average doubling time of 20 to 60 min [101]. Consequently, the sample may have already been contaminated beyond saving before antimicrobials are reintroduced with the culture medium. This hypothesis may be validated in further studies quantifying bacterial load in the supernatant after enzyme digestion. Adding antimicrobials to the enzyme solution would require careful selection, as many antimicrobials interfere with the function of enzymes such as collagenase I [102,103].

This also shows that, although very effective in comparison to established methods, the modified decontamination and disinfection protocol [17,27] may benefit from further refinement. With the current protocol, the samples are stored at 4 °C. While this is a very effective temperature for preserving cell viability, gentamycin and penicillin/streptomycin nearly completely lose their efficacy, since bacterial growth is required for the pharmacodynamic and -kinetic processes [104,105,106,107]. Even though amphotericin B has some physical mechanisms of pore creating, it too suffers from a great reduction in effectiveness [108].

Since increasing storage temperature is not an option, as cell viability needs to be maintained, other modifications are needed. The tissue samples could be stored in several smaller sections (about 3 cm in length) as opposed to one long section, increasing the surface area for passive diffusion by the antimicrobials.

Future studies should include routine microbiological analysis of all samples, particularly when in vivo application of cells and cell banking are planned. Denys et al. proposed screening for common equine viral and bacterial pathogens [34], which becomes especially relevant for allogeneic use of uc-MSCs.

The slightly higher number of cultures lost during isolation can still be tolerated, as the NM is still financially favourable with 29.8 ± 27.7% lower costs per culture.

Even after thorough rinsing and careful stripping of main and microvessels, the final cell suspension remained heavily interspersed with erythrocytes, making cell counting impossible. Plus, despite the use of cell strainers, larger cell aggregates resembling grape-like clusters appeared under the microscope mainly in the NM; single-cell suspension was not achieved. Although neither issue appeared to interfere with cell attachment or growth, future experiments might benefit from a step of erythrolysis in the protocol [109] as well as the use of a chelating agent like EDTA in an aliquot prior to counting. This way, possible contaminations might be caught earlier on, as confusion with debris from erythrocytes will then no longer be possible. Furthermore, a standardised number of cells is seeded into flasks every time, thus increasing reproducibility. Due to these issues, no initial cell count was obtained after enzymatic digestion; therefore, no direct analysis of the initial yield prior to seeding could be performed. We were, however, able to evaluate the IPT and PDT, as well as score the extent of initial cell attachment. The IPT and PDT analysis showed faster postisolation expansion, but the current dataset does not allow us to determine whether a greater number of cells was released during digestion or whether the NM uc-MSCs proliferate intrinsically faster. Further research regarding the cellular yield after digestion of both protocols would be valuable.

As shown in Table 7, the NM reduces nightshifts and overtime to the necessary minimum at NM-T_−1_. The break point created due to the 24 h collagenase incubation means that all further work can be completed within regular operating hours. Due to the shorter incubation interval, the RM protocol misses crucial pause points, thus causing not only three nightshifts but also overtime in the shifts critical for clean and concentrated work at RM-T_0_ and RM-T_1_.

In the RM, this leads to higher regional labour costs per foal. In addition to the increased labour and material costs, two other factors need to be considered. First, the employee would not be working during regular lab hours and has to be compensated for more overtime hours with lieu time. Plus, other personnel would have to cover their regular shifts, and other experiments might get delayed due to this rescheduling.

In total, under the cost assumptions of this study, the RM costs an additional 21.4% (20.9%; 20%) in labour per foal plus 21 ± 21% in material. These operational and potential economic benefits of the NM should be weighed alongside the differences in immunophenotypic profile and contamination rate observed between the two approaches, while acknowledging the exploratory nature of this pilot study. The economic benefits require further confirmation through studies with a larger sample size.

As the population of foals and mares sampled for this study was limited to German warmbloods, it remains to be determined whether our findings apply across all horse breeds. Many studies either use a mix of breeds, undeclared breeds or warmbloods [17,22,26,30,34].

In summary, the optimised protocol (NM) offers a promising alternative to established protocols, facilitating workflow flexibility and integration into standard laboratory routines even across multiple shifts and employees. These operational adjustments reduce the cost of material and labour on average, particularly regional overtime compensation. The low contamination rate in comparison to the majority of existing protocols, coupled with the faster postisolation expansion of the NM uc-MSCs, makes it a valid candidate for future studies.

## 5. Conclusions

The novel isolation method (NM) for equine uc-MSCs represents a promising alternative to the reference method (RM) that improves both laboratory and economic efficiency. Although non-inferiority was not demonstrated for some MSC-associated markers and for the exploratory trilineage differentiation endpoints, an advantage of the NM uc-MSCs is their faster PDT and IPT, which reduced the total culture time by 32.8% on average and thus resulted in a reduction of material costs per sample. Furthermore, the introduction of strategic break points reduced the labour burden from three nightshifts to one, thus lowering regional labour costs considerably. While the NM had a higher contamination rate than the RM, it remains well below rates reported in other studies and, under the economic assumptions applied in this pilot study, the additional cost associated with contaminated cultures did not offset the estimated material and labour savings.

Ultimately, the NM provides a flexible and potentially more cost-effective approach that integrates well into standard laboratory routines while maintaining a contamination rate below that of most reported isolation protocols for equine umbilical-cord-derived mesenchymal stem cells. In summary, the present pilot study delivers a strong basis for future studies and protocol adaptations.

## Figures and Tables

**Figure 1:**
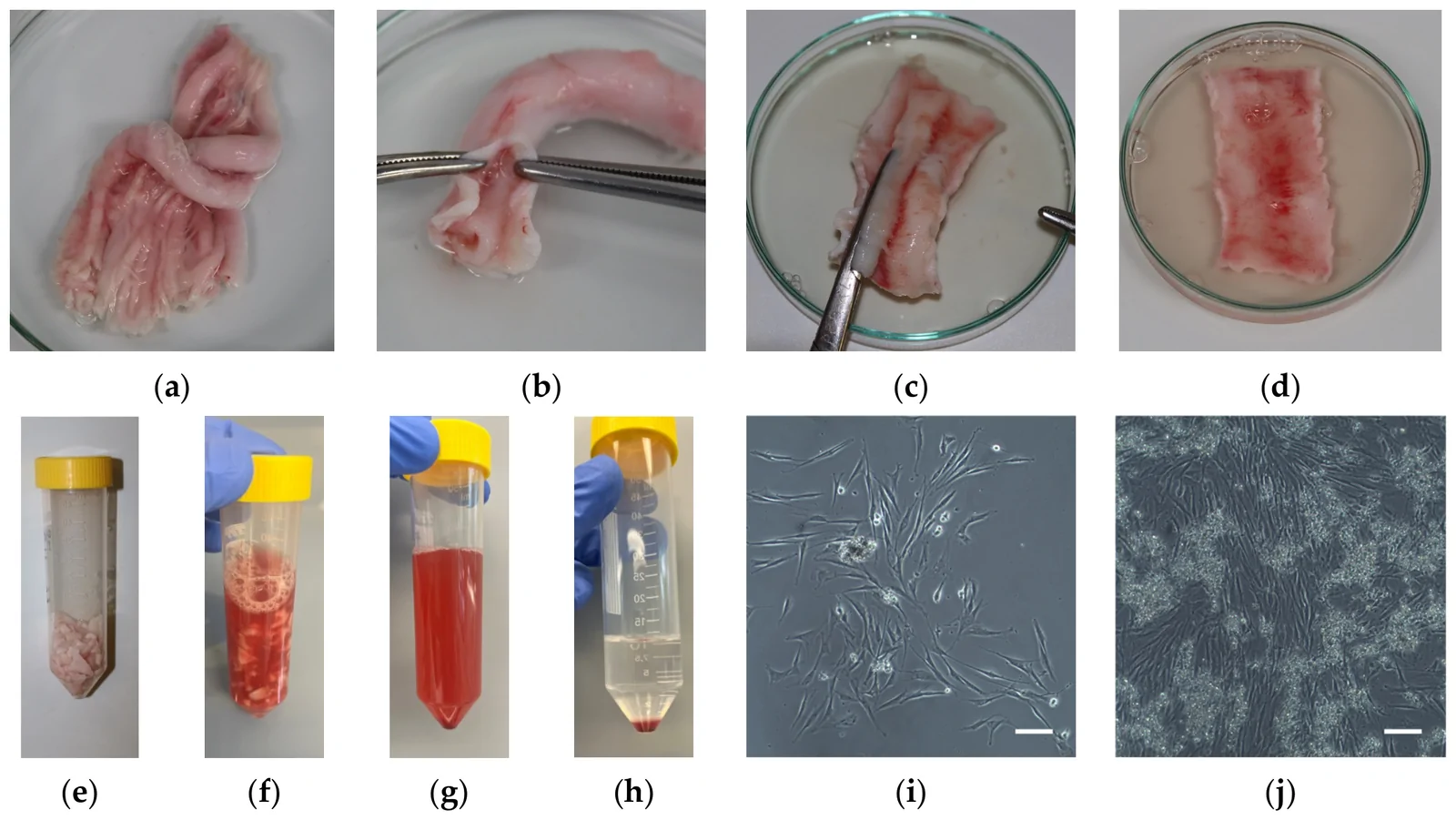
Umbilical-cord-derived mesenchymal stem cell (uc-MSC) isolation process: (**a**) Sample after decontamination; (**b**–**d**) Stripping of Wharton’s jelly (WJ); (**e**) Minced WJ before collagenase I digestion; (**f**) Samples after 3 h digestion (reference method—RM); (**g**) Samples after 24 h digestion (novel method—NM); (**h**) Cell pellet after 3-step washing; (**i**) RM uc-MSCs after 7 d of culture; (**j**) NM uc-MSCs after 7 d of culture; (**i**,**j**) Scale bar = 100 µm.

**Figure 2:**
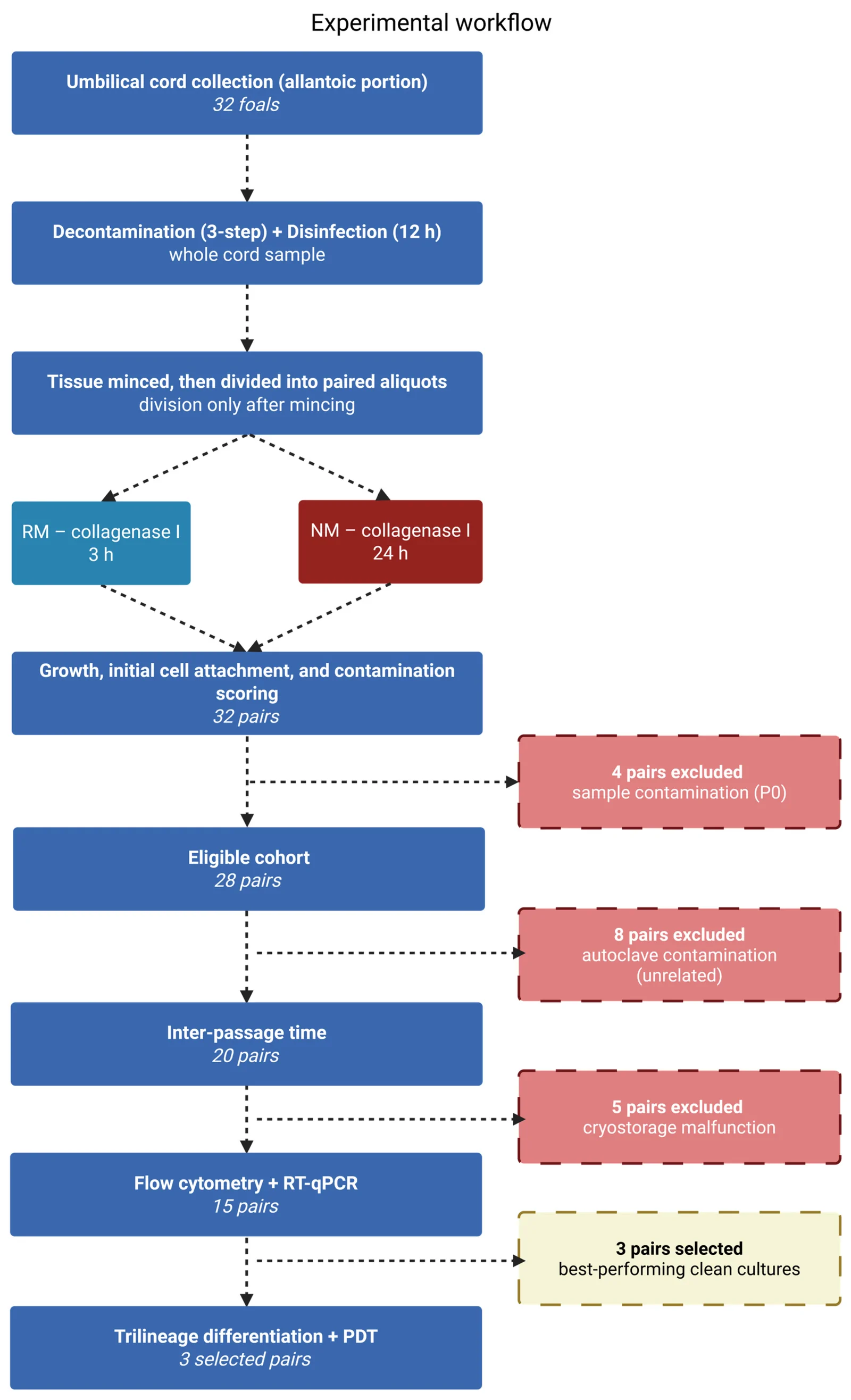
Experimental workflow diagram displaying the number of samples collected, processed, and used for further comparisons of the 3 h collagenase I incubation (reference method—RM) and the 24 h collagenase I incubation (novel method—NM). *N* = 32 umbilical cord samples were collected, decontaminated, and minced, aliquoted for either the RM or NM digestion. After initial growth scoring, paired samples from *N* = 4 foals were excluded due to microbial contamination within passage 0 (P0). Paired samples from *N* = 8 foals were excluded after an unrelated autoclave contamination. Inter-passage time (IPT) was scored for paired samples from *N* = 20 foals and after the loss of paired samples from *N* = 5 foals due to cryostorage malfunctions, paired samples from *N* = 15 foals were used for flow cytometry and RT-qPCR. Paired samples from *N* = 3 foals were selected based on RT-qPCR and flow cytometry results and were then used for all trilineage differentiations and the analysis of population-doubling time (PDT). Created in BioRender. Hollender, A. (2026) https://BioRender.com/snz35os.

**Figure 3:**
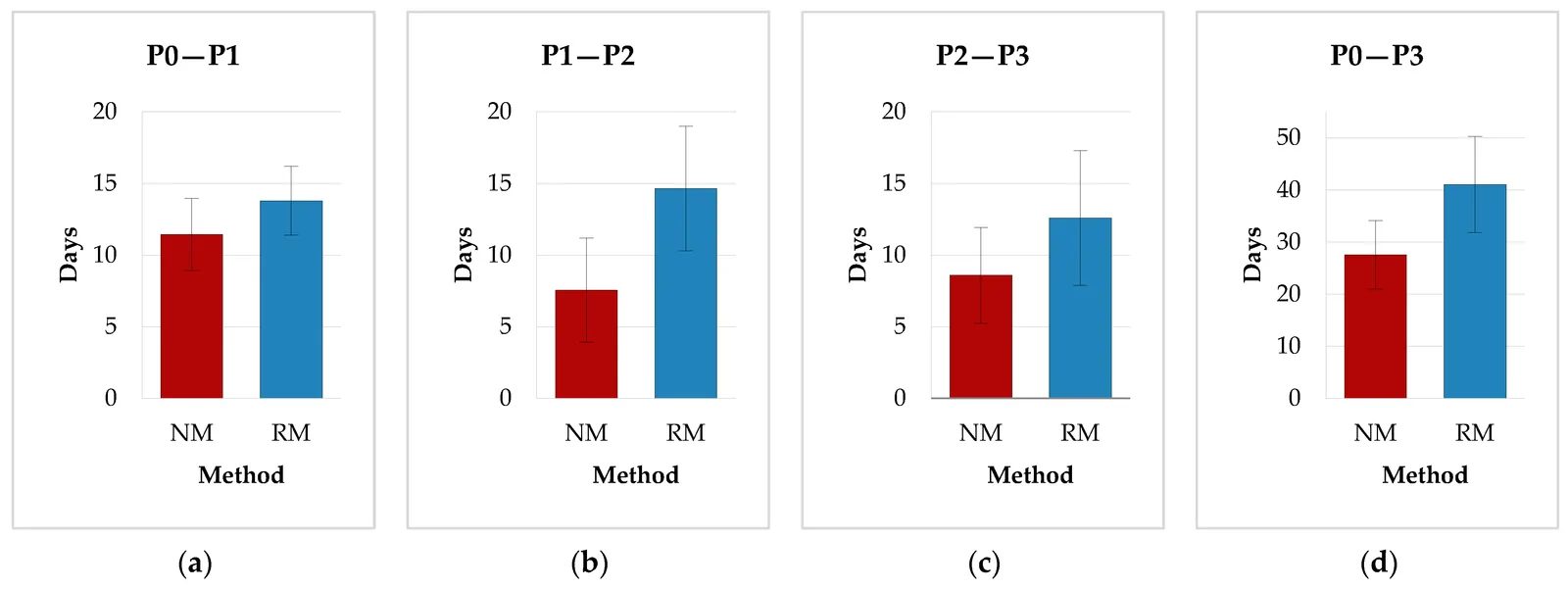
Inter-passage time (IPT) in days (d) from passage 0 (P0) to passage 3 (P3) comparing the reference method (RM), a 3 h collagenase I incubation, and the novel method (NM), a 24 h collagenase I incubation; displaying means ± standard deviations: (**a**) IPT for P0 to P1 comparing RM and NM; (**b**) IPT for P1 to P2 comparing RM and NM; (**c**) IPT for P2 to P3 comparing RM and NM; (**d**) IPT for P0 to P3 comparing RM and NM.

**Figure 4:**
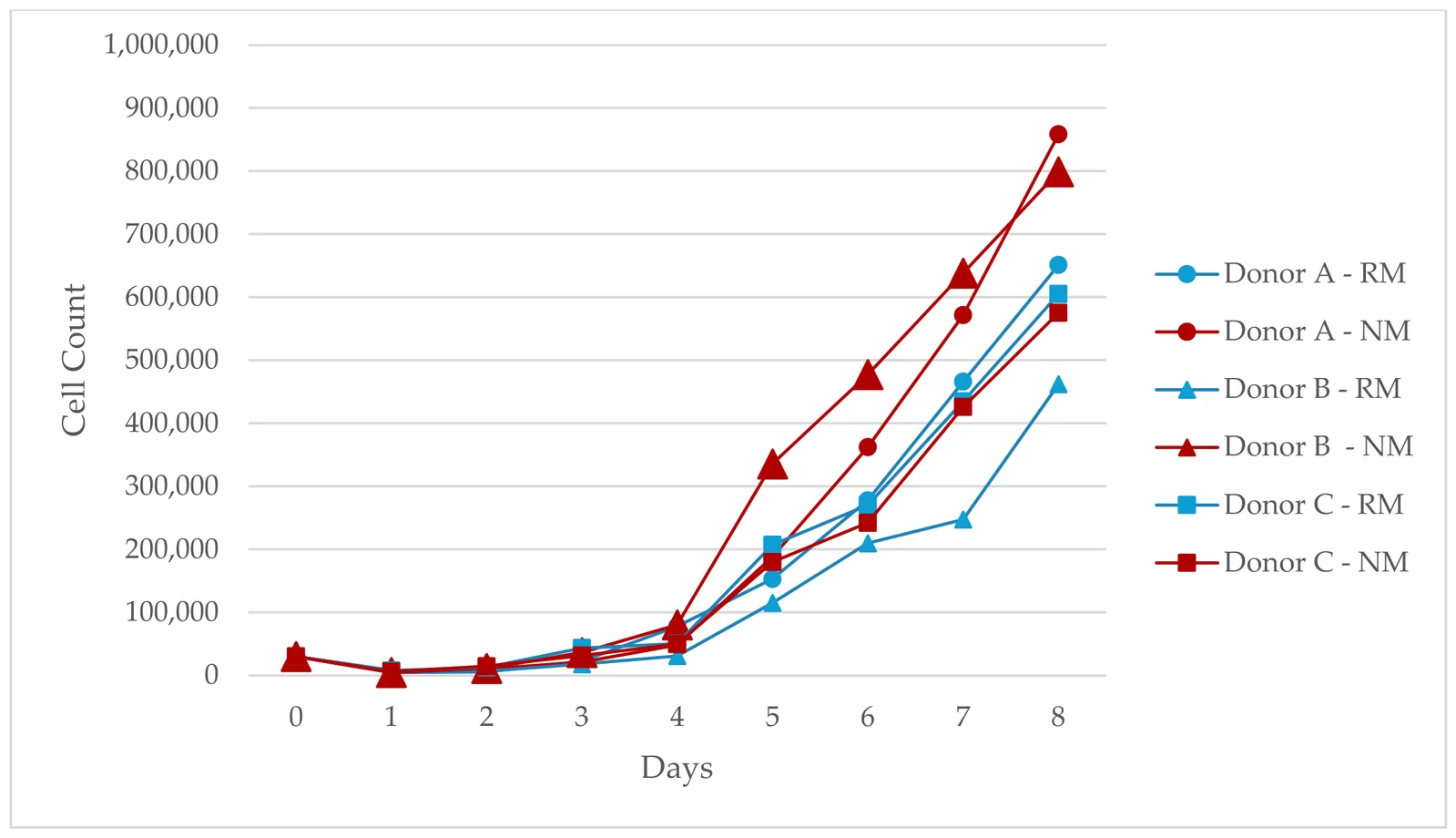
Cell counts from the population-doubling time assay (PDT), comparing the reference method (RM), a 3 h collagenase I incubation, and the novel method (NM), a 24 h collagenase I incubation. Values are means of technical replicates for each donor per day and method. RM is displayed in blue, NM in red.

**Figure 5:**
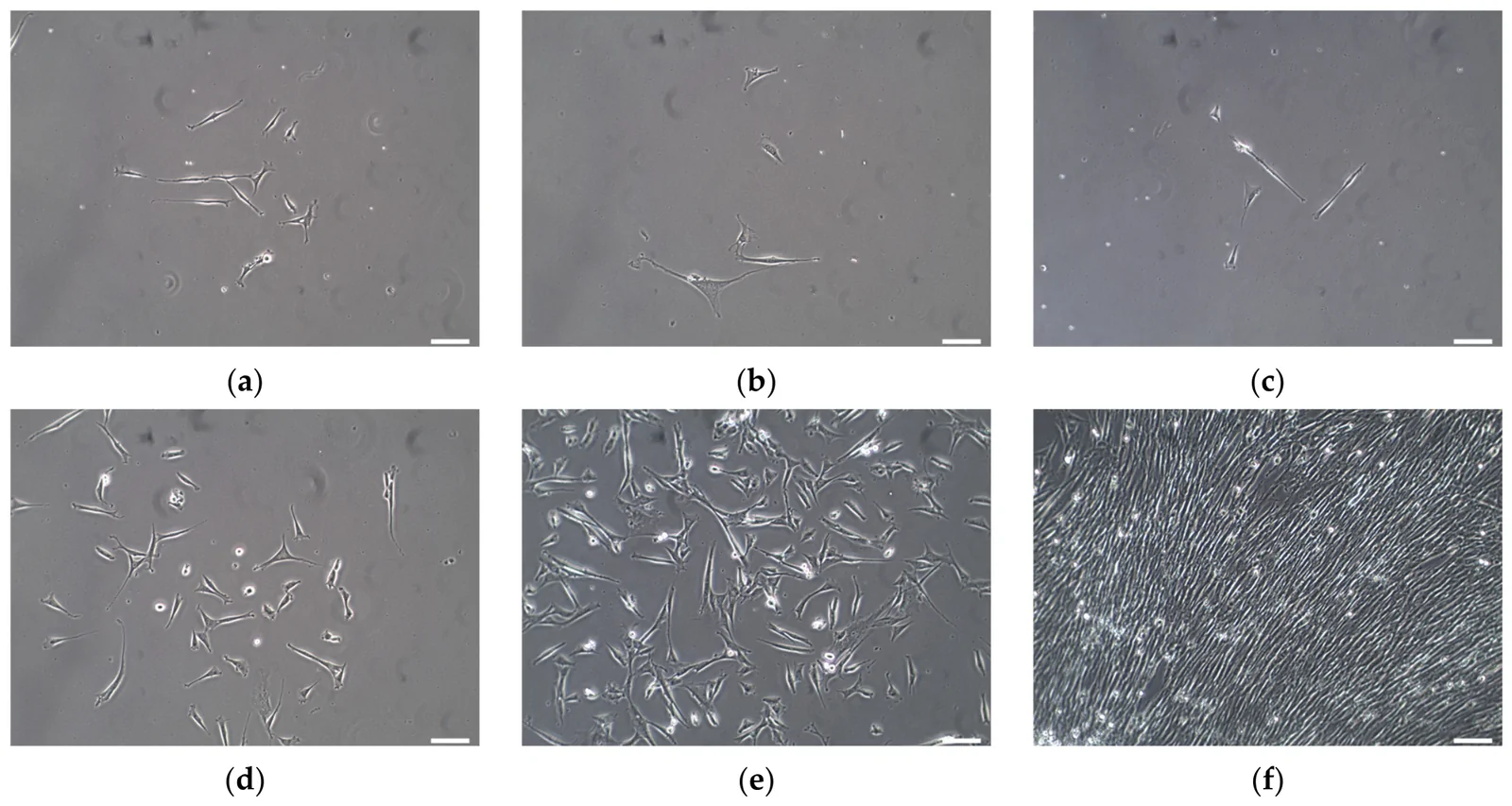
Umbilical-cord-derived mesenchymal stem cell (uc-MSC) morphology directly after isolation in passage 0 (P0). Rapidly self-renewing cells (RS cells), spindle-shaped cells (SS cells), and flattened cells (FC cells) [75]. Scale bar = 100 µm. (**a**) SS cells; (**b**) FC cells; (**c**) SS and RS cells; (**d**) RS, SS, and FC cells; (**e**) 50% confluence and all three cell types; (**f**) 100% confluence and only SS cells visible, end of P0.

**Figure 6:**
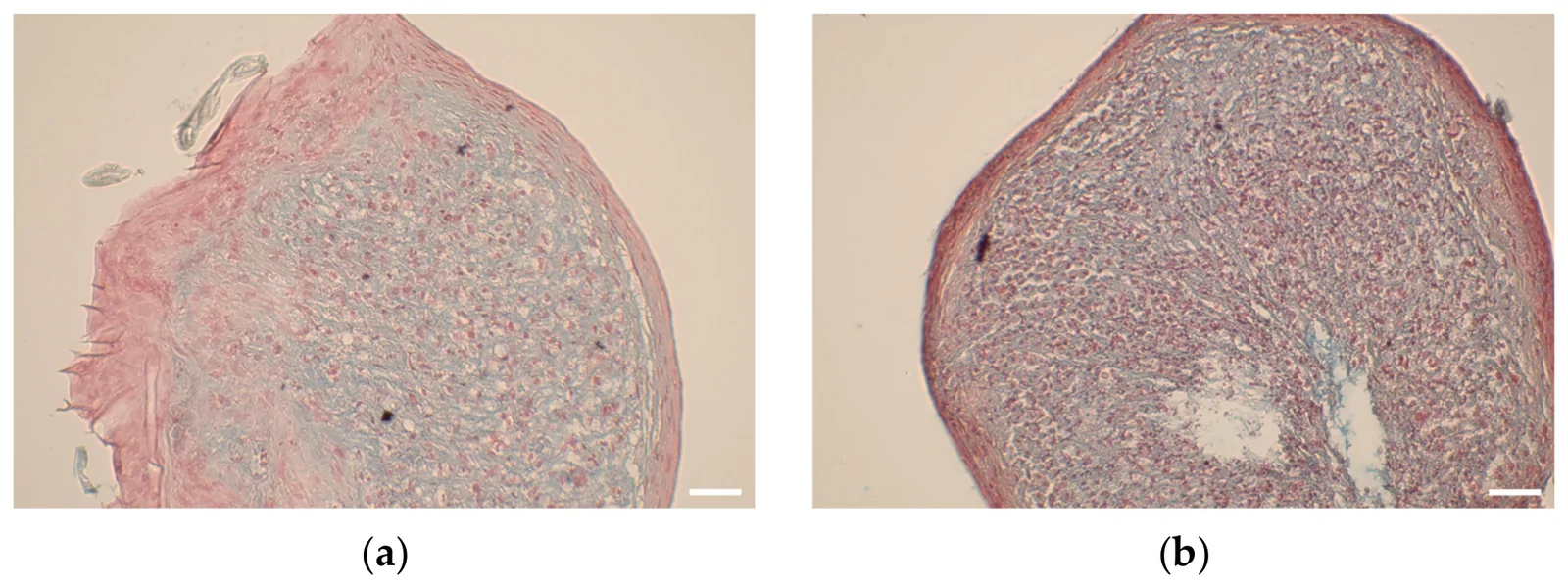
Chondrogenic differentiation of umbilical-cord-derived mesenchymal stem cell pellets (uc-MSCs). Microscopy images after nuclear fast red and Alcian blue staining. Comparison of the reference method (RM), a 3 h collagenase I incubation, and the novel method (NM), a 24 h collagenase I incubation. Scale bar = 100 µm. (**a**) RM (3 h) sample after nuclear fast red (pink) and Alcian blue (blue); (**b**) NM sample after nuclear fast red (pink) and Alcian blue (blue).

**Figure 7:**
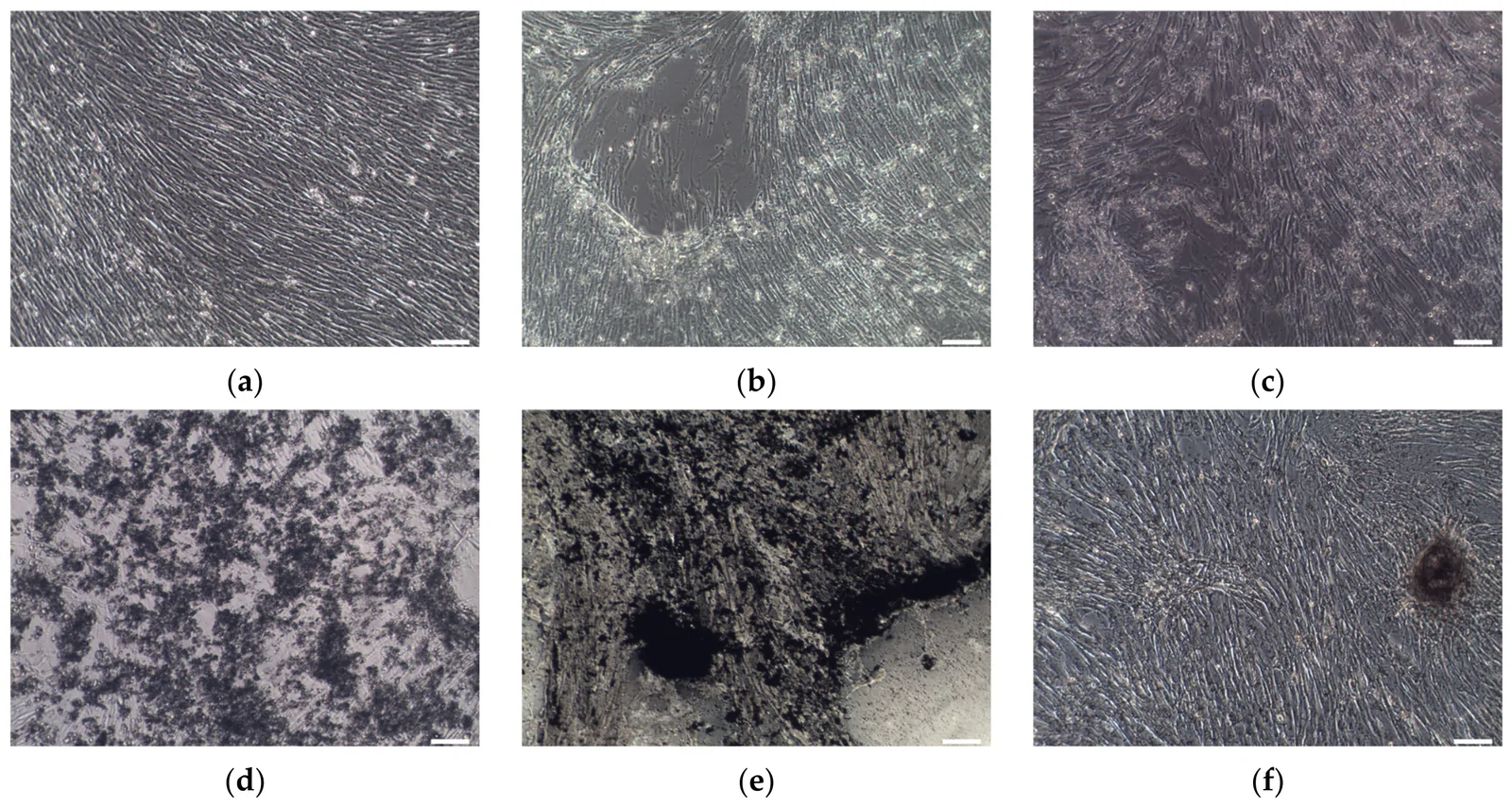
Osteogenic differentiation of umbilical-cord-derived mesenchymal stem cells (uc-MSCs). Microscopy images after von Kossa staining. Comparison of the reference method (RM), a 3 h collagenase I incubation, and the novel method (NM), a 24 h collagenase I incubation. Scale bar = 100 µm. (**a**) Day 0 (T_0_) unstained; (**b**) day 7 (T_7_) unstained; (**c**) day 14 (T_14_) unstained; (**d**) day 20 (T_20_) unstained; (**e**) T_20_ after von Kossa staining; (**f**) T_20_ negative control (NC) after von Kossa staining.

**Figure 8:**
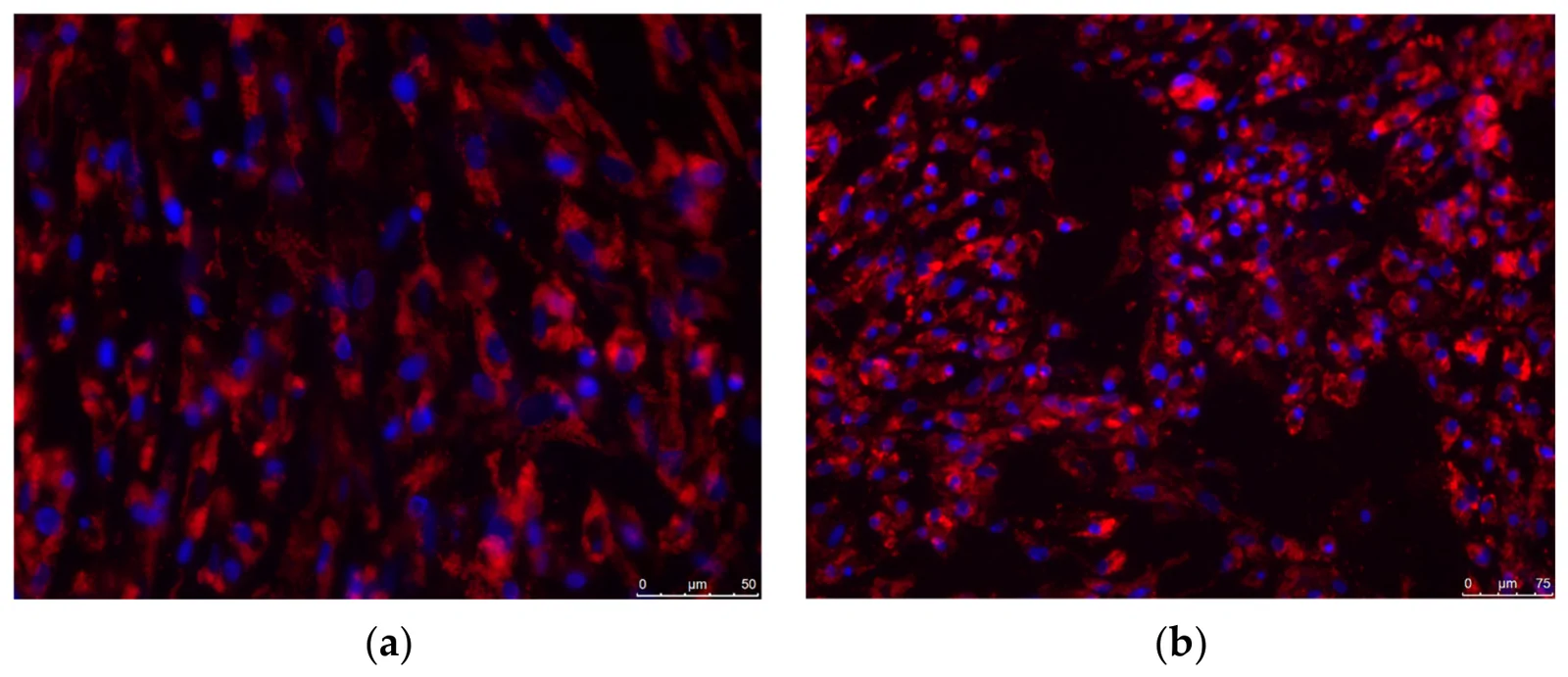
Adipogenic differentiation of umbilical-cord-derived mesenchymal stem cells (uc-MSCs). Fluorescence microscopic images after Nile red and DAPI staining. Comparison of the reference method (RM), a 3 h collagenase I incubation, and the novel method (NM), a 24 h collagenase I incubation. Measured at 480/530 nm. (**a**) RM sample after DAPI (blue) and Nile red staining (red); (**b**) NM sample after DAPI (blue) and Nile red staining (red).

**Figure 9:**
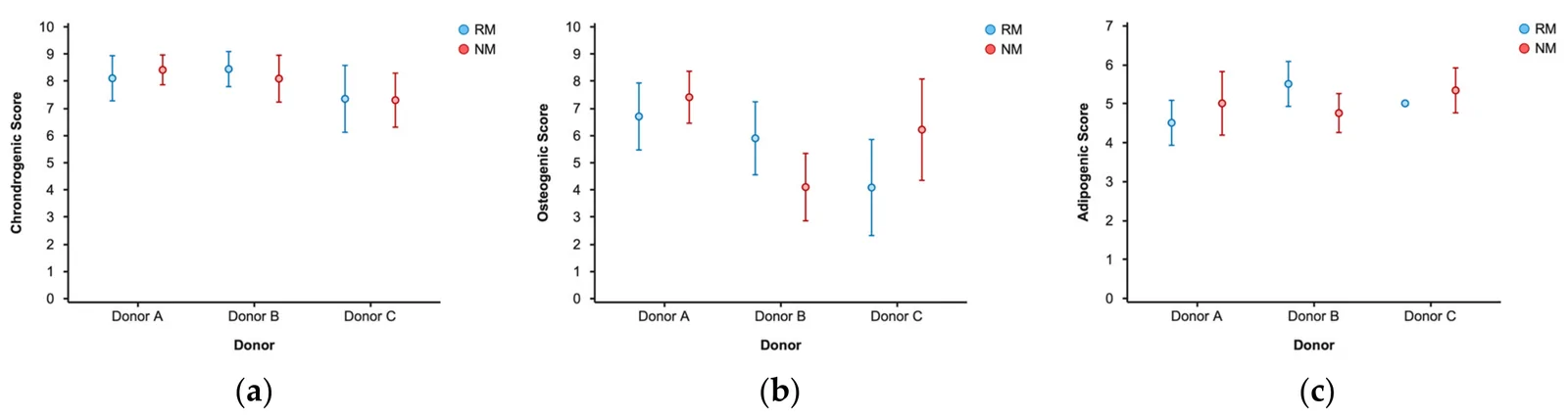
Donor-level paired data for each trilineage differentiation score. Data for each donor are presented as the mean ± standard deviation. Created with BioRender Graphing (BioRender, Toronto, ON, Canada; https://www.biorender.com). (**a**) The chondrogenic differentiation score was assessed on a scale from 0 to 9 points [55]. (**b**) The osteogenic differentiation score was assessed on a scale from 0 to 9 points. (**c**) The adipogenic differentiation score was assessed on a scale from 0 to 6 points [30].

**Table 1:** Semi-quantitative osteogenic differentiation score.

% of Field of View Covered by Mineral Deposits (10×)		Size of Predominant Variety of Mineral Deposits (10×)		Arrangement and Clustering of Mineral Deposits (10×)	
0	0–5%	0	No mineral deposits visible or non-specific silver precipitation (dark brown instead of clear black deposits)	0	No mineral deposits visible or non-specific silver precipitation (dark brown instead of clear black deposits)
1	>5–30%	1	Small punctuate mineral deposits (<size of cell nucleus)	1	Deposits scattered across field of view, minimal aggregation
2	>30–70%	2	Micronodules (=size of the cell nucleus, <size of the cell)	2	Deposits scattered across field of view, singular smaller clusters visible
3	>70–100%	3	Nodules (>size of cell nucleus, ≥size of the cell)	3	Deposits mainly aggregated as large 3D calcified clusters

**Table 2:** Primers and gene function.

Gene	Function	Accession No.	Forward Sequence 5′–3′
			Reverse Sequence 3′–5′
*ACTB*	Reference gene	NM_001081838.1	GCCAACCGCGAGAAGATGAC
			AGTCCATCACGATGCCAGTG
*GAPDH*	Reference gene	NM_001163856.1	AAGAAGGTGGTGAAGCAGG
			GCATCGAAGGTGGAAGAGTGGG
*RN18s*	Reference gene	NW_019643269.1	ACTCACACGGGAAACCTCAC
			AACCAGACAAATCGCTCCAC
*DENND4A*	Stem cell gene	XM_023655064.1	TGCAACCTCTCTTTTTGCC
			CGCATTTCACCATTCTTGTC
*DNMT3B*	Stem cell gene	XM_023626323.1	ACTTGGTGATTGGTGGAAGC
			ACACTCCAGGAACCGAGAGA
*MYC*	Stem cell gene	XM_001497991.1	CAGCGACTCTGAAGAAGAAC
			ACTGTCCAACTTAGCCCTC
*POU5F1/OCT4*	Stem cell gene	XM_001490108	AGCAATTTGCCAAGCTCC
			GTCTCTGCTTTGCATATCTCC
*THY-1*	Stem cell gene	XM_023644995.1	CCAGCAAGTACAACATCAAGG
			CTTATGCCCTCGCACTTGAC

**Table 3:** Flow cytometry antibodies.

Positive/Negative Markers	Antigen	Host Species	Target Species	Clone	Stain	Company	Product No.	Concentration
+	CD29	Mouse—IgG1	human	TS2/16	APC	Milteny Biotec	130-101-271	1:100
−	CD34	Mouse—IgG1	human	581	PE	BioLegend	343505	1:100
+	CD44	Rat—IgG2b	human/mouse	IM7	eFluor 450	eBioscience	48-0441-82	1:250
−	CD45	Mouse—IgG2a	human	F10-89-4	Biotin	Bio-Rad	MCA87B	1:10
+	CD90.1	Mouse—IgG2a	rat/mouse	HIS51	APC-Fluor 780	BioLegend	47-0900-82	1:200
+	CD90.1	Mouse—IgG1	rat/mouse	OX-7	Alexa Fluor 450	BioLegend	202521	1:200
+	CD90.1	Mouse—IgG1	rat/mouse	OX-7	Alexa Fluor 700	BioLegend	202527	1:200
+	CD90.2	Rat—IgG2b	mouse	30-H12	PerCP	BioLegend	105322	1:200
+	CD105	Mouse—IgG1	human	SN6	PE-Cyanine 7	eBioscience—Invitrogen	25-1057-41	1:100
**Function**		**Stain**		**Company**		**Product No.**		**Concentration**
Secondary antibody anti-biotin		FITC		eBioscience– Invitrogen		11-4317-87		1:200
Viability antibody		eFluor 506		eBioscience– Invitrogen		65-0866-18		1:1000

**Table 4:** Non-inferiority null and alternative hypotheses; prespecified non-inferiority margin.

Method	Hypothesis	Margin of Non-Inferiority
PDT	(H_0_): µ_NM_ − µ_RM_ ≥ 12 h	+12 h
	(H_1_): µ_NM_ − µ_RM_ < 12 h	
IPT	(H_0_): µ_NM_ − µ_RM_ ≥ 7 d	+7 d
	(H_1_): µ_NM_ − µ_RM_ < 7 d	
Trilineage– chondrogenic	(H_0_): µ_NM_ − µ_RM_ ≤ −1.5 points	−1.5 points
	(H_1_): µ_NM_ − µ_RM_ > −1.5 points	
Trilineage– adipogenic	(H_0_): µ_NM_ − µ_RM_ ≤ −1.0 points	−1.0 points
	(H_1_): µ_NM_ − µ_RM_ > −1.0 points	
Trilineage– osteogenic	(H_0_): µ_NM_ − µ_RM_ ≤ −1.5 points	−1.5 points
	(H_1_): µ_NM_ − µ_RM_ > −1.5 points	
RT-qPCR	(H_0_): µ_NM_ − µ_RM_ ≥ 0.2345	+0.2345
	(H_1_): µ_NM_ − µ_RM_ < 0.2345	
Flow cytometry—positive markers	(H_0_): µ_NM_ − µ_RM_ ≤ −15%	−15%
	(H_1_): µ_NM_ − µ_RM_ > −15%	
Flow cytometry—negative markers	(H_0_): µ_NM_ − µ_RM_ ≥ 15%	+15%
	(H_1_): µ_NM_ − µ_RM_ < 15%	

**Table 5:** Reverse-Transcription quantitative PCR results for the reference method (RM) and the novel method (NM); results of the non-inferiority analysis, *N* = 15; prespecified margin = +0.2345 ΔCt.

Gene	Group (RM or NM)	Paired Difference	Mean (ΔCt Values)	Standard Deviation	Lower Confidence Interval (95%)	Upper Confidence Interval (95%)	*p* Value (α = 0.05)
*DENND4A*	RM	−0.30 (−0.71 to 0.11)	9.89	0.58	9.57	10.21	0.028
	NM		9.59	0.65	9.23	9.95	
*DNMT3B*	RM	−0.09 (−0.36 to 0.18)	11.99	0.46	11.74	12.25	0.034
	NM		11.91	0.35	11.71	12.10	
*MYC*	RM	−0.45 (−0.84 to −0.07)	8.59	0.63	8.25	8.94	0.005
	NM		8.14	0.43	7.90	8.38	
*POU5F1/* *OCT4*	RM	0.26 (−0.28 to 0.81)	14.00	0.80	13.56	14.44	1.00
	NM		14.26	0.92	13.75	14.77	
*THY1*	RM	1.16 (0.41 to 1.91)	6.29	0.64	5.93	6.64	1.00
	NM		7.45	1.29	6.73	8.16	

**Table 6:** Flow cytometry results from the reference method (RM) and the novel method (NM); results of the non-inferiority analysis, *N* = 15; prespecified margin = ±15%. The paired difference is the Hodges–Lehmann estimate with its 95% confidence interval, corresponding to the Wilcoxon signed-rank test.

Antigen	Group (RM or NM)	Paired Difference (Hodges–Lehmann)	Median (% Expression)	IQR (25th–75th)	25th Percentile	75th Percentile	*p* Value (α = 0.05)
CD29	RM	−13.49 (−37.90; −0.10)	95.7	7	91.2	98.2	0.466
	NM		92.6	43.8	53.3	97.1	
CD34	RM	−1.90 (−19.00; 1.15)	1.0	2.8	0	2.8	0.001
	NM		0.1	1	0	1	
CD44	RM	−10.50 (−27.65; 1.95)	98.5	2.1	97.5	99.6	0.147
	NM		98.1	27.2	72.7	99.9	
CD45	RM	−3.25 (−19.70; 1.50)	2.5	10.4	0.7	11.1	<0.001
	NM		1.5	5.4	0.5	5.9	
CD105	RM	−0.10 (−1.50; 1.70)	0.3	0.8	0.2	1	0.015
	NM		0.2	0.8	0	0.8	

**Table 7:** Comparison of workflow and shift distribution from the reference method (RM) and the novel method (NM).

Method	Details	Day −1 (T_−1_)	Day 0 (T_0_)	Day 1 (T_1_)	Day 2 (T_2_)
Reference Method (3-Hour Incubation)	Start of shift	19:00	10:15	14:30	-
	End of shift	05:00	22:30	23:30	-
	Tasks	Sampling	Enzymatic digestion and seeding of cells	Washing of cells	-
Novel Method (24-Hour Incubation)	Start of shift	19:00	10:15	11:00	11:30
	End of shift	05:00	16:00	17:00	17:30
	Tasks	Sampling	Enzymatic digestion	Seeding of cells	Washing of cells

## Data Availability

The raw dataset presented in this article may be provided from the Institute of Veterinary Physiology of Freie Universität Berlin (Jörg Aschenbach) or the Equine Clinic of Freie Universität Berlin (Heidrun Gehlen) upon reasonable request. Patient data that might reveal the identity of the sampled foals/mares is not available due to privacy reasons. All other data are included in the manuscript and its Supplementary Materials. Parts of this work were presented as a poster at the 13th PhD and Doctoral Student Symposium of the Doctoral Students of Dahlem Research School, School of Veterinary Medicine, Freie Universität Berlin, Germany on 10 October 2024.

## Supplementary Materials

The following supporting information can be downloaded at: https://www.mdpi.com/article/10.3390/ani16203258/s1; Table S1: Disposable materials; Table S2: Reusable utensils; Table S3: Software; Table S4: Instruments; Table S5: Reagents and chemicals; Table S6: Results of the non-inferiority analysis for the inter-passage time (IPT) assay in days comparing the reference method (RM) and the novel method (NM); Table S7: Results of the non-inferiority analysis for the population-doubling time (PDT) assay from day 1 to day 6 (T1-T6) excluding plateau growth phase in hours comparing the reference method (RM) and the novel method (NM); Figure S1: Representative flow cytometry plots and gating strategies for umbilical-cord-derived mesenchymal stem cells (uc-MSCs); Listing S1; R-Code for Hodges–Lehmann effect estimates and 95% confidence intervals; Listing S2: R-Code for Holm multiple testing correction.

## Abbreviations

The following abbreviations are used in this manuscript:

Amp B	Amphotericin B
FBS	Foetal bovine serum
FC	Flattened cell [75]
FOV	Field of view
FSC	Forward light scatter
Genta	Gentamycin
IPT	Inter-passage time
MHC Class II	Major histocompatibility complex class II
MSCs	Mesenchymal stem cells
NC	Negative control
NM	Novel method
PBS (+/+)	PBS with Ca^2+^ and Mg^2+^
PBS (−/−)	PBS without Ca^2+^ and Mg^2+^
PDT	Population-doubling time
Pen/Strep	Penicillin/Streptomycin
RM	Reference method
-RT-control	No-reverse-transcriptase control
RS	Rapid self-renewing cell [75]
SS	Spindle-shaped cell [75]
SSC	Sideward light scatter
uc-MSCs	Umbilical cord derived mesenchymal stem cells
WJ	Wharton’s jelly

## Appendix A

**Table A1:** Retail pricing and cost-efficiency analysis (as of 28 July 2026).

Product Name	Size	Price (in €)	Source
Penicillin–streptomycin	100 mL	35.80	[58]
Amphotericin B	300 mL	160.89	[59]
Gentamycin	100 mL	379.00	[63]
DMEM low g	500 mL	27.80	[64]
Foetal bovine serum	500 mL	580.00	[62]
Collagenase 1	500 mg	201.00	[60]
TPP conical tube 50 mL	360 pieces	360.00	[61]
PBS tablets	100 pieces	188.00	[65]
Ethanol 100%	10 L	569.00	[66]
Braunol 7.5%	1 L	25.41	[67]

**Table A2:** Labor costs and pay grades in Germany for federal employees [68,69].

Legislation	Pay Grade	Experience Level	Gross Wage (per h)	Overtime Pay (in %)	Nighttime Pay (in %)
TVöD	E5	1	18.42 €	30%	20%
	E6	1	19.11 €	30%	20%
	E7	1	19.43 €	30%	20%
	E8	1	20.56 €	30%	20%
	E9a	1	21.58 €	30%	20%
	E9b	1	22.29 €	30%	20%
	E9c	1	23.65 €	30%	20%
	E10	1	24.32 €	15%	20%
	E11	1	25.18 €	15%	20%
	E12	1	26.04 €	15%	20%
	E13	1	28.90 €	15%	20%
	E14	1	31.24 €	15%	20%
TV-L	E5	1	18.04 €	30%	20%
	E6	1	18.70 €	30%	20%
	E7	1	18.98 €	30%	20%
	E8	1	20.06 €	30%	20%
	E9a	1	21.24 €	30%	20%
	E9b	1	21.24 €	30%	20%
	E10	1	23.69 €	30%	20%
	E11	1	24.51 €	15%	20%
	E12	1	25.29 €	15%	20%
	E13	1	27.92 €	15%	20%
	E14	1	30.18 €	15%	20%
