# Supplementary material for: Refining the Protocol for Equine Umbilical-Cord-Derived Mesenchymal Stem Cell Isolation: A Pilot Study on Resource Efficiency and Cellular Properties
Source: Animals (Basel). 2026 Oct 16;16(20):3258. doi: 10.3390/ani16203258 (PMC13700546; doi:10.3390/ani16203258)
Supplement: Supplementary file 1 [file animals-16-03258-s001.zip › animals-4506164-supplementary.pdf]

Supplementary Material – Table S1, S2, S3, S4, S5, S6, S7, Figure S1, and Listing S1, S2:

Table S1. Disposable materials.

Product Name	Size	Manufacturer	Product No.
Tissue culture flasks	T25	TPP Techno Plastic Products AG, Trasadingen, Switzerland	90026
Tissue culture flasks	T75	TPP Techno Plastic Products AG, Trasadingen, Switzerland	90076
Tissue culture test plates	6-well	TPP Techno Plastic Products AG, Trasadingen, Switzerland	92006
Tissue culture test plates	12-well	TPP Techno Plastic Products AG, Trasadingen, Switzerland	92012
Tissue culture test plates	24-well	TPP Techno Plastic Products AG, Trasadingen, Switzerland	92024
CytoOne® multiwell plates	24-well	Starlab GmbH, Hamburg, Germany	CC7682-7524
Microplate, 96-well, PP, v-bottom	96-well	Greiner Bio-One, Kremsmünster, Austria	651261
Centrifuge tube	15 mL, PP	TPP Techno Plastic Products AG, Trasadingen, Switzerland	91015
Centrifuge tube	50 mL, PP	TPP Techno Plastic Products AG, Trasadingen, Switzerland	91050
Syringe filter	PES, 0.22 µm	TPP Techno Plastic Products AG, Trasadingen, Switzerland	99722
Syringe filter	PES, 0.45 µm	TPP Techno Plastic Products AG, Trasadingen, Switzerland	99745
Cell strainer	70 µm	Corning Inc., Corning, NY, USA	431751
Nunc™ serological pipette tips with filter	5 mL	Thermo Fisher Scientific Inc., Waltham, MA, USA	170373N
Nunc™ serological pipette tips with filter	10 mL	Thermo Fisher Scientific Inc., Waltham, MA, USA	170374N
Nunc™ serological pipette tips with filter	25 mL	Thermo Fisher Scientific Inc., Waltham, MA, USA	170375N
epTIPS®	50 to 1,000 µL	Eppendorf SE, Hamburg, Germany	0030000927
epTIPS®	2 to 200 µL	Eppendorf SE, Hamburg, Germany	0030000870
epTIPS®	0.1 to 10 µL	Eppendorf SE, Hamburg, Germany	0030000811
Cryogenic vials	2 mL tubes	VWR International GmbH, Darmstadt, Germany	479-1262
Cell Counting Slides for TC10™/TC20™ Cell Counter	Dual-Chamber, 30 slides, 60 counts	Bio-Rad Laboratories GmbH, Feldkirchen, Germany	#1450011

Table S2. Reusable utensils.

Product Name	Type/Size	Manufacturer	Product No.
Drain strainer	mesh stainless steel	Fackelmann GmbH Co. KG, Hersbruck, Germany	-
Serological pipette	pipetus® 100-240 Volt	Hirschmann® Laborgeräte GmbH & Co. KG, Eberstadt, Germany	9907200
Research Plus, Eppendorf	0.5 to 10 µL	Eppendorf SE, Hamburg, Germany	3123000020
Research Plus, Eppendorf	10 – 100 µL	Eppendorf SE, Hamburg, Germany	3123000047
Research Plus, Eppendorf	100 – 1,000 µL	Eppendorf SE, Hamburg, Germany	3123000063
Mr. Frosty™	1 to 2 mL tubes	Thermo Fisher Scientific Inc., Waltham, MA, USA	5100-0001

Table S3. Software.

Product Name	Manufacturer	Version
FACSDiva	BD Biosciences, Milpitas, CA, USA	V 8
FlowJo software	Tree star Inc., Ashland, OR, USA	Version 10 & 11
NCSS 2026 Statistical Software (2026)	NCSS, LLC. Kaysville, UT, USA	NCSS 2026
ImageFocus	Euromex Microscopen bv, Duiven, Netherlands	V 4.0
Images Plus	MOTICEUROPE, S.L.U., Barcelona, Spain	V 3.1
Leica Application Suite (LAS) AF	Leica Microsystems GmbH, Wetzlar, Germany	Version 3.2.0.9652
R	R Foundation	V 4.6.1
BioRender	Science Suite Inc	Online version on 09.09.2026

Table S4. Instruments.

Product Name	Manufacturer	Product No.
AE2000 microscope	MOTICEUROPE, S.L.U., Barcelona, Spain	11722298
Agilent-Bioanalyzer-Nano	Agilent RNA 6000 Nano Kit, Agilent Technologies, Böblingen, Germany	G2939BA
BD FACSCanto II	BD Biosciences, Milpitas, CA, USA	338960
BR-2000 Vortexer, 120 V	Bio-Rad Laboratories GmbH, Feldkirchen, Germany	#1660610EDU
Centrifuge 5418 R	Eppendorf SE, Hamburg, Germany	5401000010
Centrifuge 5804R	Eppendorf SE, Hamburg, Germany	5805000010
CO_2 incubator CB 160	BINDER GmbH, Tuttlingen, Germany	4064343307000
DC.500c camera	Euromex Microscopen bv, Duiven, Netherlands	DC.5000c
DFC 350 FX Leica camera	Leica Microsystems GmbH, Wetzlar, Germany	12730047
DMI 6000B/AF6000 fluorescence microscope	Leica Microsystems GmbH, Wetzlar, Germany	-
Epredia™ HM 325 Rotary Microtome	Thermo Fisher Scientific Inc., Waltham, MA, USA	23-900-668
Grant Instruments™ PHMT Thermoshaker for Microtubes and Microplates	Grant Instruments (Cambridge) Ltd, Royston, United Kingdom	15201176
Logos One	A. Menarini Diagnostics, Berlin, Germany	-
M6 camera	MOTICEUROPE, S.L.U., Barcelona, Spain	1100600101111
Mastercycler® nexus X2 - PCR Thermal Cycler	Eppendorf SE, Hamburg, Germany	6337000019
Nanophotometer™	Implen, GmbH, Munich, Germany	P 330
TC20™	Bio-Rad Laboratories GmbH, Feldkirchen, Germany	#1450102EDU
ViiA7 Thermocycler	Applied Biosystems/Life Technologies; Foster City, CA, USA	4453536
VWB2 12	VWR International, Radnor, PA, USA	462-0557

Table S5. Reagents and chemicals.

Product Name	Type/Size	Manufacturer	Product No.
Accutase®	100 mL	Carl Roth GmbH + Co. KG, Karlsruhe, Germany	1Y18.1
Alcian blue-core red	100 mL	Morphisto GmbH, Offenbach am Main, Germany	13416
Amphotericin B 250 µg/mL	50 mL	Corning Inc., Corning, NY, USA	15333671
Braunol 7.5% povidone-iodine antiseptic solution	1 L	B. Braun Vet Care GmbH, Tuttlingen, Germany	7612449002557 and 01588716
Corning™ ITS+ Premix Universal Culture Supplement	20 mL	Corning Inc., Corning, NY, USA	11593560
Corning™ Ultrapure Laminin, Mouse	1 mg	Corning Inc., Corning, NY, USA	10419592
DMSO	50 mL	AppliChem GmbH, Darmstadt, Germany	A3672,0050
Eosin, Conc. Watery 2%	10 L	Waldeck GmbH & Co. KG, Münster, Germany	2C-140.10000
Fetal Bovine serum	500 mL	Sigma-Aldrich®, Merck KGaA, Darmstadt, Germany	F7524-500ML
Gentamycin sulphate solution	100 mL	Carl Roth GmbH + Co. KG, Karlsruhe, Germany	HN09.2
Gibco™, Collagenase, type I, powder	500 mg	Thermo Fisher Scientific Inc., Waltham, MA, USA	17100017
Gibco™ DMEM, high glucose, pyruvate	500 mL	Thermo Fisher Scientific Inc., Waltham, MA, USA	11995065
Gibco™ DMEM, low glucose, GlutaMAX™ Supplement, pyruvate	500 mL	Thermo Fisher Scientific Inc., Waltham, MA, USA	21885025
Gibco™ Dulbecco's phosphate-buffered saline without Mg^{2+} and Ca^{2+} (DPBS [-/-])	500 mL	Thermo Fisher Scientific Inc., Waltham, MA, USA	14190094
Gibco™, StemPro™ Adipogenesis Differentiation Kit	1 kit	Thermo Fisher Scientific Inc., Waltham, MA, USA	A1007001
Gibco™, Trypsin-EDTA (0.25%), phenol red	100 mL	Thermo Fisher Scientific Inc., Waltham, MA, USA	25200056
Invitrogen™, Rabbit Serum	5 mL	Thermo Fisher Scientific Inc., Waltham, MA, USA	PLN5001
iScript™ cDNA Synthesis Kit	25 x 20 µL	Bio-Rad Laboratories GmbH, Feldkirchen, Germany	1708890
NucleoSpin RNA II, Mini Kit for RNA Isolation	50 preps	Macherey Nagel GmbH & Co. KG, Düren, Germany	740955.50
Penicillin-Streptomycin, 100x	100 mL	Sigma-Aldrich®, Merck KGaA, Darmstadt, Germany	P4333-100ML
ROTI®Cell Dulbecco's phosphate-buffered saline with Mg^{2+} and Ca^{2+} (DPBS [+/+])	1 L	Carl Roth GmbH + Co. KG, Karlsruhe, Germany	9131.1
ROTI®Histofix 4%	500 mL	Carl Roth GmbH + Co. KG, Karlsruhe, Germany	P087.4
Silver Nitrate 5 %	250 mL	Morphisto GmbH, Offenbach am Main, Germany	10375.00250

Trypan blue	50 mL	Sigma-Aldrich®, Merck KGaA, Darmstadt, Germany	93595-50ML
Trypsin-EDTA (0,25%), phe-nolred	100 mL	Thermo Fisher Scientific Inc., Waltham, MA, USA	25200056

Table S6. Results of the non-inferiority analysis for the inter-passage time (IPT) assay in days comparing the reference method (RM) and the novel method (NM). Passage 0 to passage 1 (P_0–P_1), passage 1 to passage 2 (P_1–P_2), and passage 2 to passage 3 (P_2–P_3). N = 20; pre-specified margin = +7 d.

Passage	Group (RM or NM)	Paired Difference	Mean (ΔCt Values)	Standard Deviation	Lower Confidence Interval (95%)	Upper Confidence Interval (95%)	p Value (α = 0.05)
P_0–P_1	RM	–2.35 (–4.16 to –0.54)	13.80	2.40	12.68	14.92	< 0.001
	NM		11.45	2.52	10.27	12.63	
P_1–P_2	RM	–7.10 (–9.30 to –4.90)	14.65	4.34	12.62	16.68	< 0.001
	NM		7.55	3.63	5.85	9.25	
P_2–P_3	RM	–4.00 (–7.03 to –0.97)	12.60	4.71	10.40	14.80	< 0.001
	NM		8.60	3.35	7.03	10.17	

Table S7. Results of the non-inferiority analysis for the population-doubling time (PDT) assay from day 1 to day 6 (T_1-T_6) excluding plateau growth phase in hours comparing the reference method (RM) and the novel method (NM). N = 3; pre-specified margin = +12 h.

Assay	Group (RM or NM)	Paired Difference	Mean (ΔCt Values)	Standard Deviation	Lower Confidence Interval (95%)	Upper Confidence Interval (95%)	p Value (α = 0.05)
PDT	RM	–3.11 (–13.05 to 6.82)	23.23	2.65	16.64	29.83	0.045
	NM		20.12	2.23	14.59	25.65	

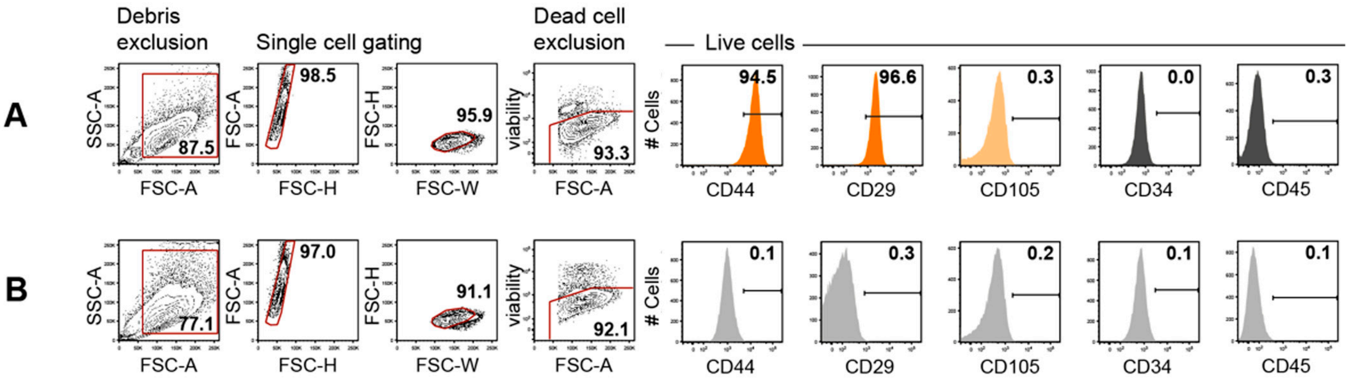

Figure S1. Representative flow cytometry plots and gating strategies for umbilical cord-derived mesenchymal stem cells (uc-MSCs). A: Exemplary results obtained after staining uc-MSCs with specific antibodies to mesenchymal stem cells markers. B: Paired unstained control uc-MSCs. Subcellular debris was excluded based on low particle size (low forward light scatter, FSC) and low sideward light scatter (SSC). After removal of potential doublets based on FSC properties, dead cells were excluded using a viability dye. The fluorochrome signals reporting the expression of the given surface markers are shown as histograms for the gated live cells. The stained cells homogenously expressed the two mesenchymal stem cell markers CD44 and CD29.

Listing S1. R-Code for Hodges-Lehmann effect estimates and 95% confidence intervals after Wilcoxon-signed rank tests. Version V 4.6.1 (Table S3)

```
library(readxl)

daten <- read_excel(file.choose())

for (m in c("CD29", "CD34", "CD44", "CD45", "CD105")) {

  x <- daten[[paste(m, "- 24h")]]      # NM

  y <- daten[[paste(m, "- 3h")]]       # RM

  w <- wilcox.test(x, y, paired = TRUE, conf.int = TRUE, exact = FALSE)

  cat(sprintf("%-6s    HL = %6.2f      95%% CI [%6.2f, %6.2f]\n",

              m, w$estimate, w$conf.int[1], w$conf.int[2]))

}
```

Listing S2. R-Code for Holm multiple testing correction. Version V 4.6.1 (Table S3)

```
# Multiple testing correction - same as R's stats::p.adjust
p <- c(0, 0.007, 0.125, 0.125, 0.25)
alpha <- 0.05

# Adjusted p-values for the active method
adj <- p.adjust(p, method = "holm")
adj
sum(adj <= alpha)

# Compare all six methods side by side
sapply(c("bonferroni", "holm", "hochberg", "hommel", "BH", "BY"),
       function(m) sum(p.adjust(p, m) <= alpha))
```
